# Supplementary figures and images for: Breaking spore dormancy in budding yeast transforms the cytoplasm and the solubility of the proteome
Source: PLoS Biol. 2023 Apr 20;21(4):e3002042. doi: 10.1371/journal.pbio.3002042 (PMC10118125; doi:10.1371/journal.pbio.3002042)

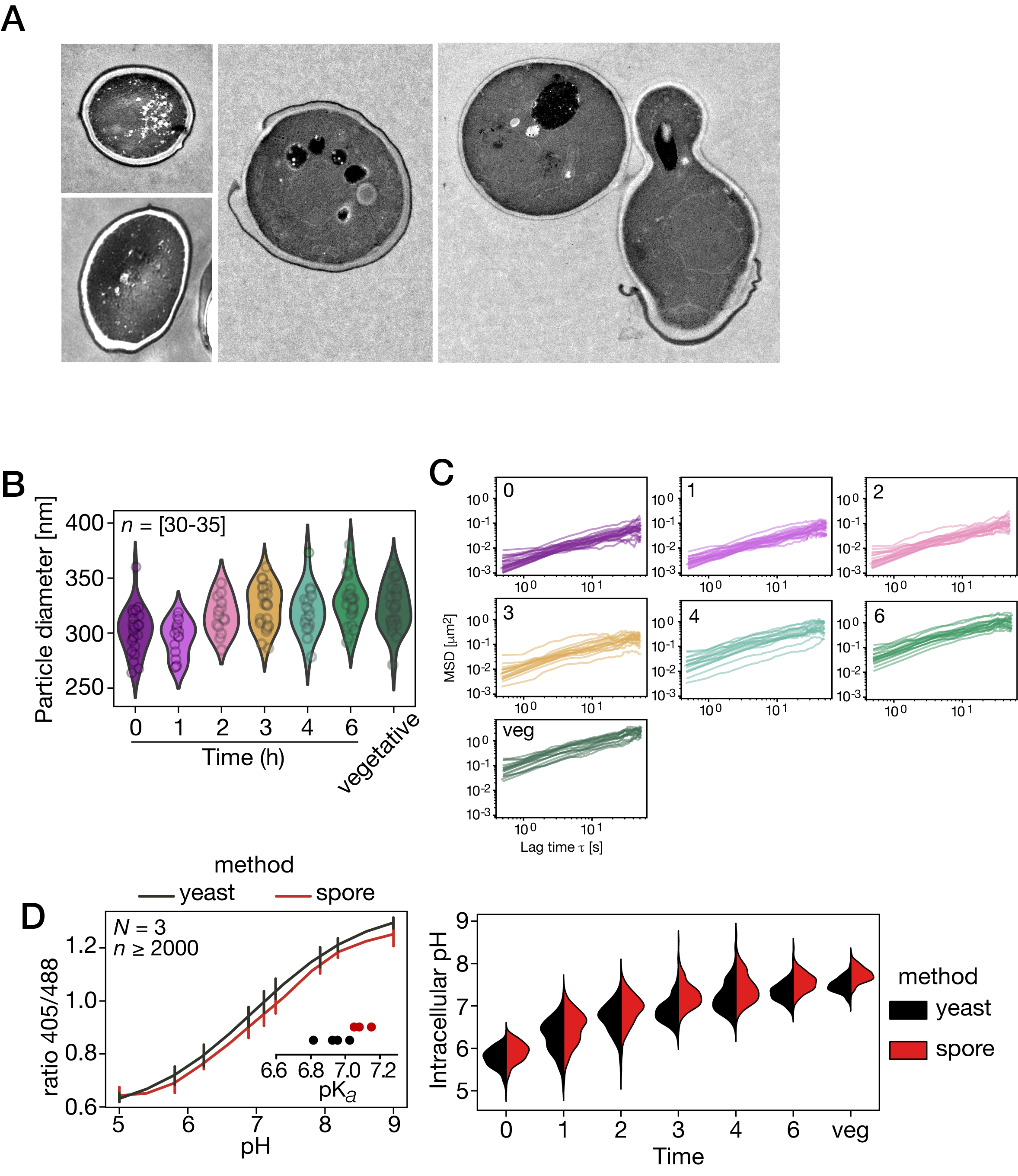

Supplement: S1 Fig — (A) Transmission electron microscopy images of spores at the indicated time after exposure to rich medium. Scale bar represents 1 μm. (B) Size of individual particles tracked at each time point during germination and in vegetatively growing cells. At least 30 particles were tracked at each time point. (C) Mean square displacement (MSD) of individual particles tracked at the indicated time after germination induction. (D) Left, intracellular pH calibration curves determined using vegetative yeast (black) or spores (red). Although the curve using spores is slightly more basic, measurement of pH in germination (right) using either curve shows that spores are acidic and that intracellular pH increases steadily during germination. Logistic function fitted to the data from vegetative cells was used for Fig 1G. Error bars represent standard deviation of 3 replicates. The data underlying this figure can be found in S1 Data. (TIF) [file pbio.3002042.s001.tif]

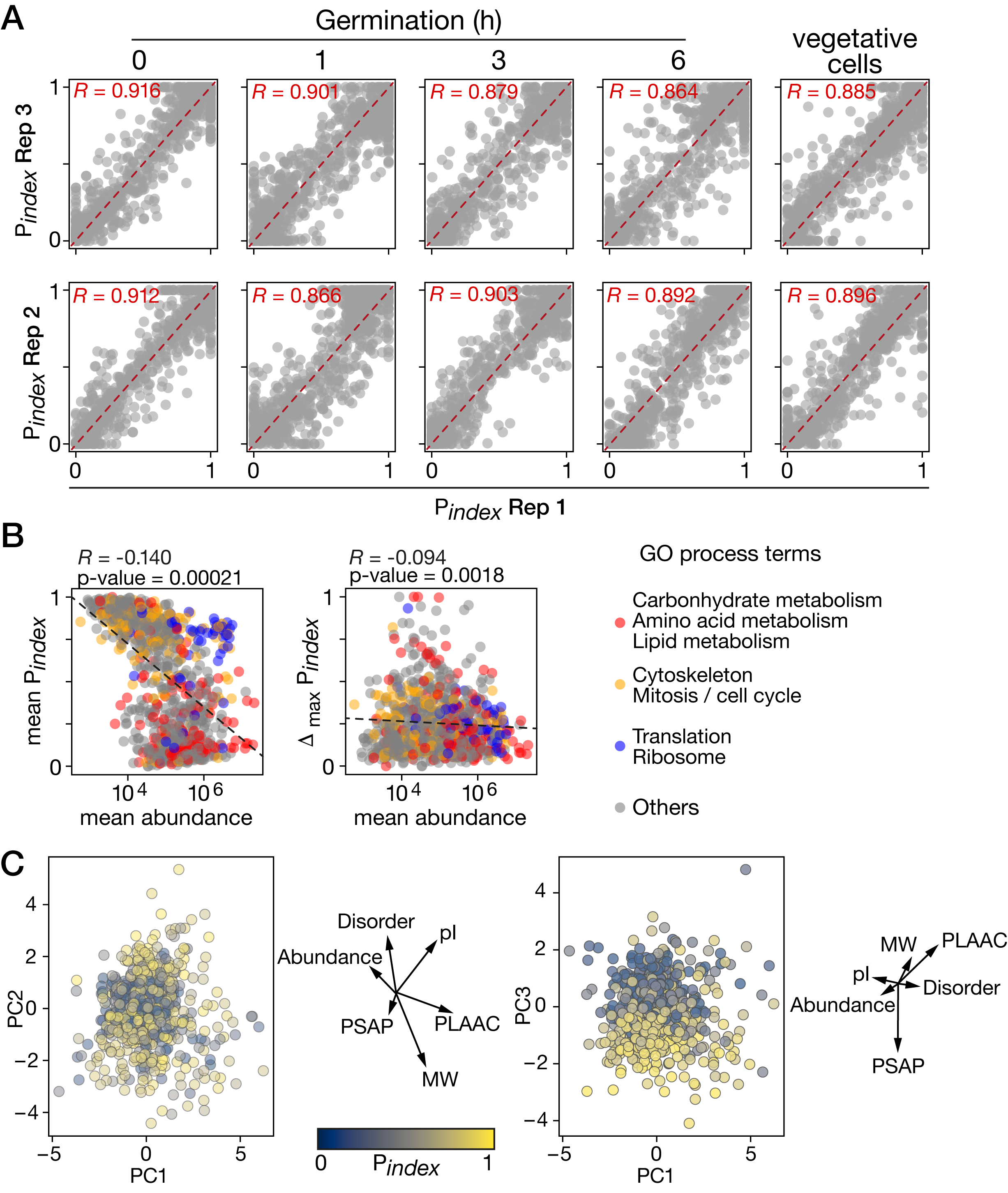

Supplement: S2 Fig — Related to Fig 2. (A) Pindex values are plotted against other replicates. Pearson’s correlation coefficients are indicated on each graph. For all correlations, p-value < 0.0001. (B) Mean of the absolute abundance estimated from mass spectrometry data during germination is plotted against mean Pindex values (left), or the maximal Pindex variation (right, ΔmaxPindex) of each protein. Points are colored depending on the GO function term. Pearson’s correlation coefficient with the log10-transformed abundance values are shown with the corresponding p-values. (C) PCA analysis of protein properties. Protein distribution across PC1 vs. PC2 (left) and PC1 vs. PC3 (right). Dots are colored according to the mean Pindex value. Beside the graph is the vector representation indicating the strength and direction of the contribution of each variable to the distribution; sequence-based estimation of molecular weight (MW) and isoelectric point (pI); mean abundance measured from our proteomic data; prion-like amino acid composition (PLAAC) prediction score; analysis and prediction score of phase separation (PSAP); sequence-based prediction of disorder (Metapredict). The data underlying this figure can be found in S2 Data. (TIF) [file pbio.3002042.s002.tif]

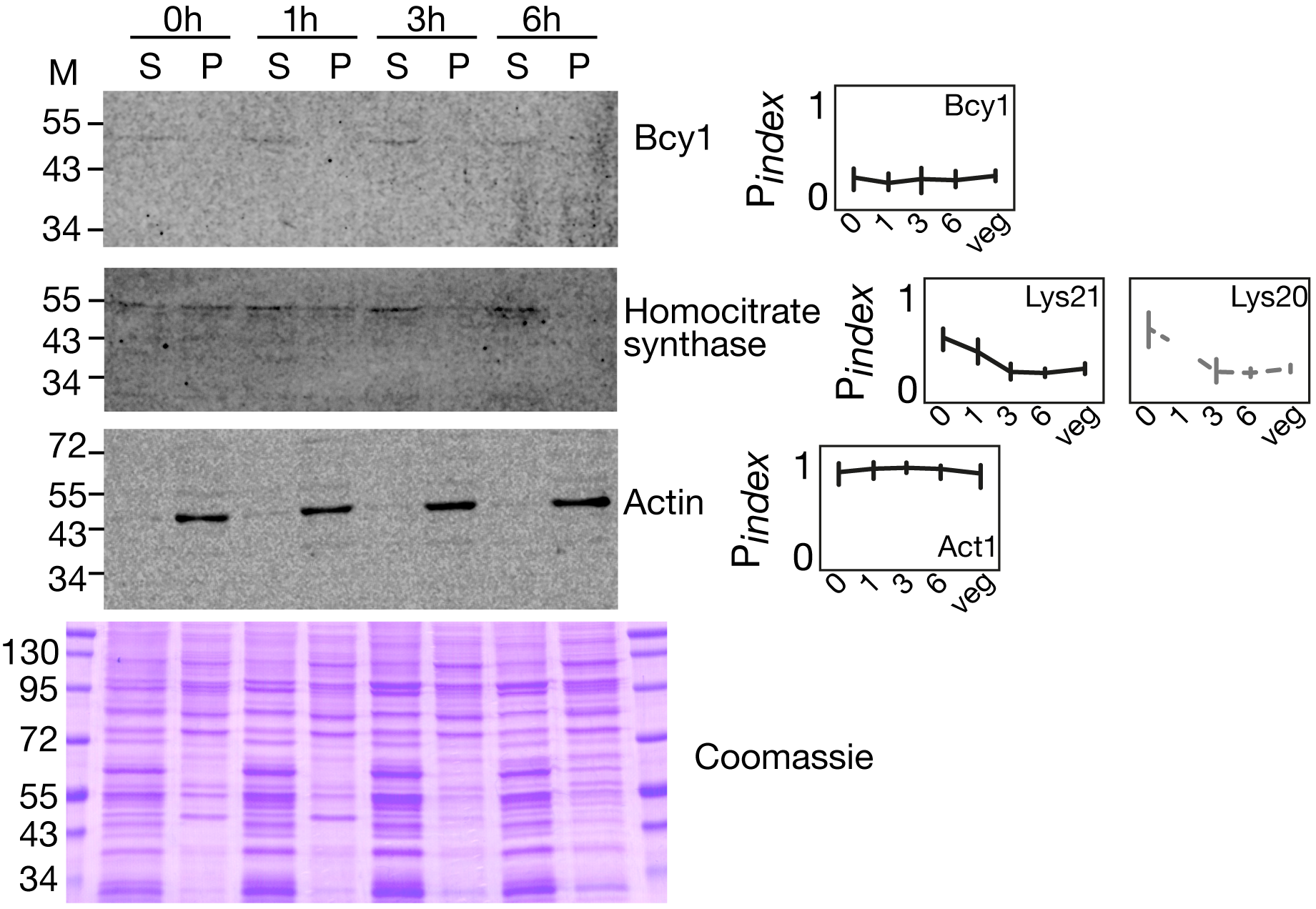

Supplement: S3 Fig — Left, the same fractionned protein extract (S, supernatant fraction; P, pellet fraction) used in MS measurements were analyzed by SDS-PAGE with antibodies that were available for yeast endogenous proteins. Shown are anti-Bcy1, anti-homocitrate synthase and anti-actin western blots, and an identically loaded gel stained by Coomassie. Protein molecular weights in the ladder (NEB# P7706) are indicated in kDa. Right, Pindex trajectories of the proteins analyzed by western blot. Homocitrate synthase isozyme Lys20 was poorly detected at 1-h time point. Error bars represent standard deviation of 3 replicates. The data underlying this figure can be found in S3 Data, and the raw blot images can be found in S1 Raw Images. (TIF) [file pbio.3002042.s003.tif]

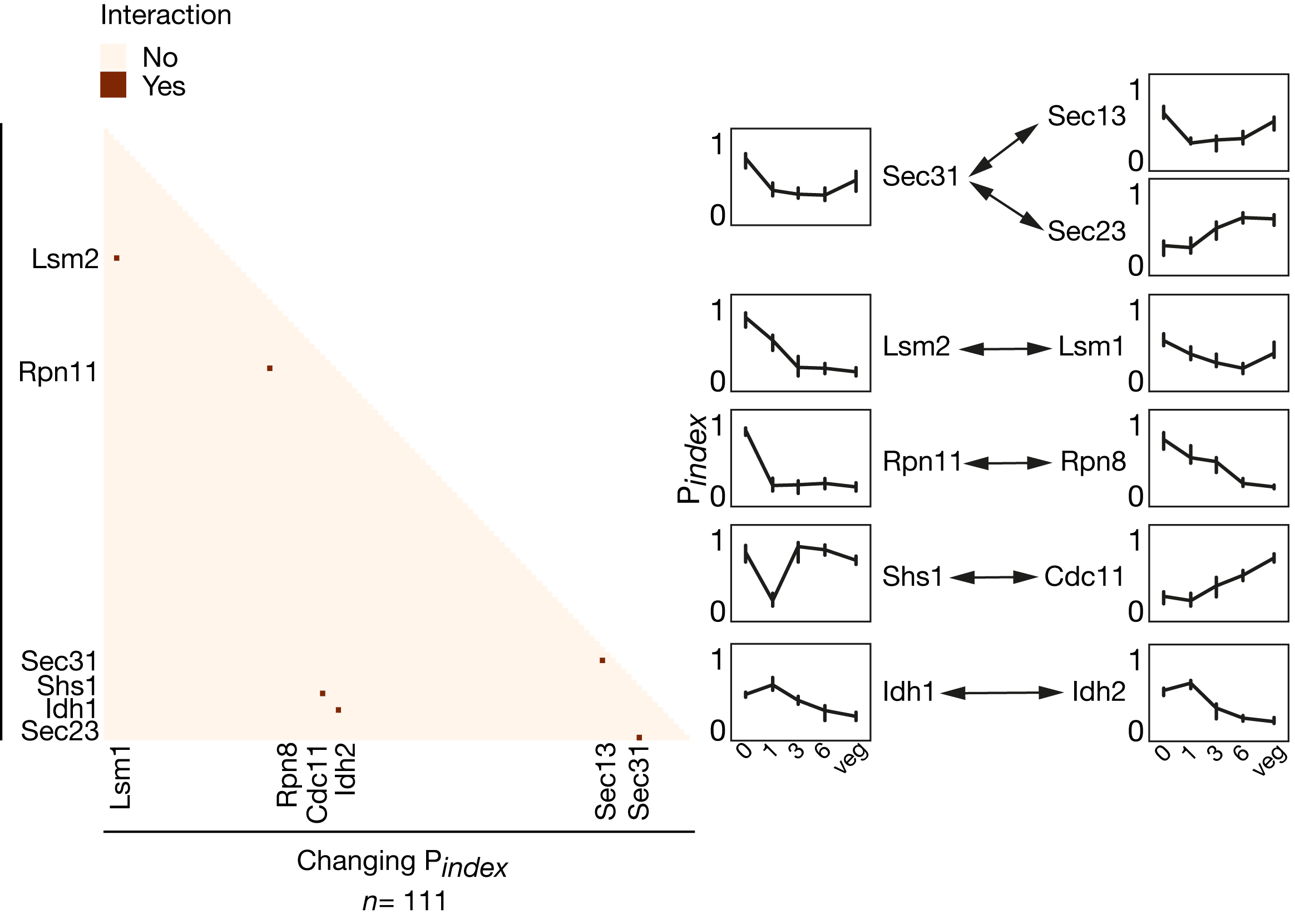

Supplement: S4 Fig — Left, interaction between the 111 Changing Pindex proteins was searched through the known physical interaction database (BioGRID v4.4.216). Pairs of interacting proteins are marked in red on the heatmap with the identity of the partners on the side. Right, the 6 pairs of interacting proteins are indicated with arrows, and the Pindex trajectories for each protein is shown. Error bars represent standard deviation of 3 replicates. The data underlying this figure can be found in S4 Data. (TIF) [file pbio.3002042.s004.tif]

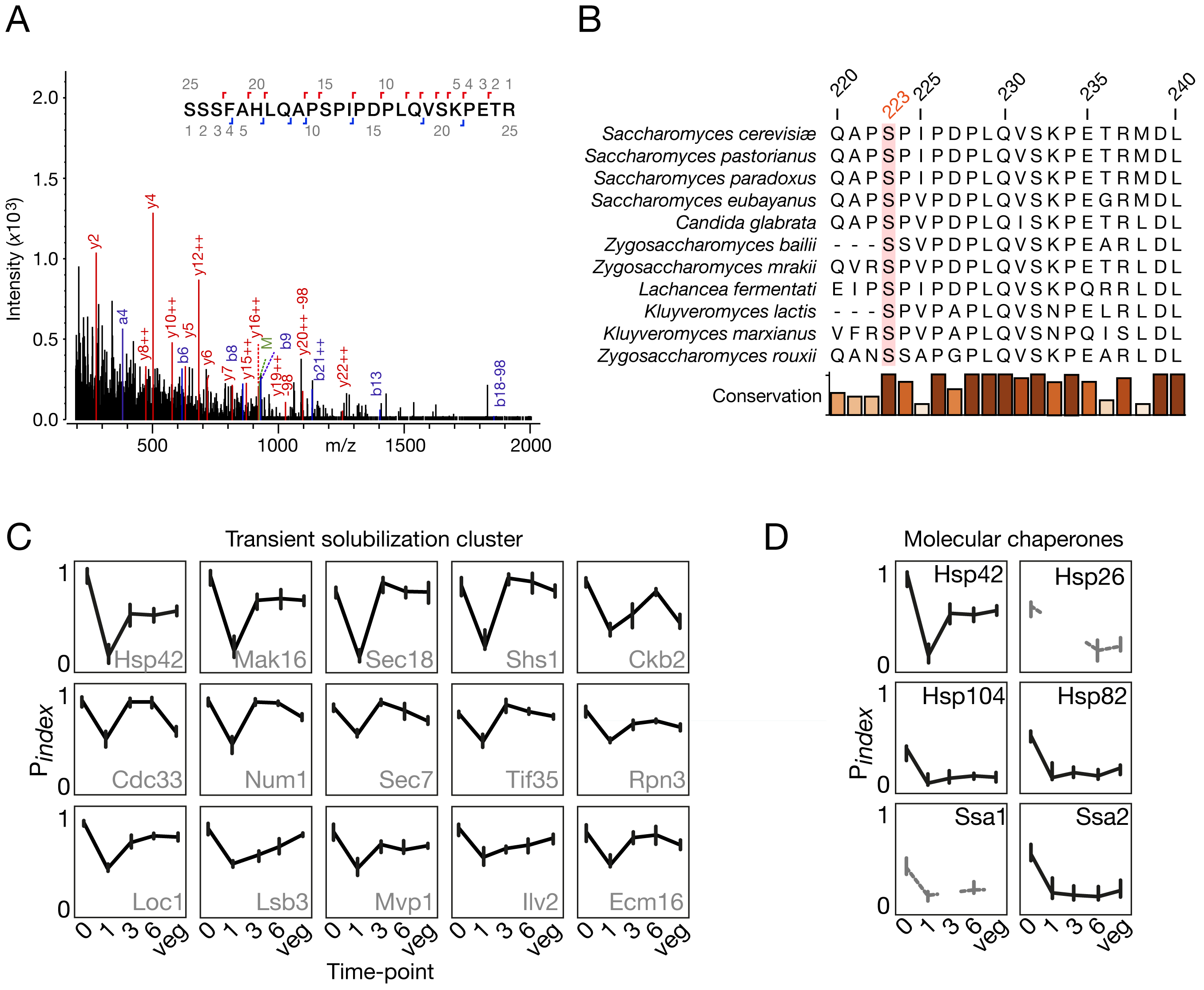

Supplement: S5 Fig — (A) MS spectra example of phosphorylated S223 peptide on Hsp42. (B) Multiple sequence alignment of Hsp42 orthologs. Numbers on top refer to residue position in the S. cerevisiae protein. S223 is underlined in orange. Relative conservation is shown with the bars at the bottom. Only a small portion of the sequences are shown. (C) Individual Pindex trajectories for each 15 proteins in the transient solubilization cluster. Error bars represent standard deviation of 3 replicates. (D) Pindex trajectories of molecular chaperones detected in our experiments. Hsp26 and Ssa1 have only partial data since they are not well detected. However, these data reveal that Hsp42 has a unique sedimentation profile among molecular chaperones. Error bars represent standard deviation of 3 replicates. The data underlying this figure can be found in S5 Data. (TIF) [file pbio.3002042.s005.tif]

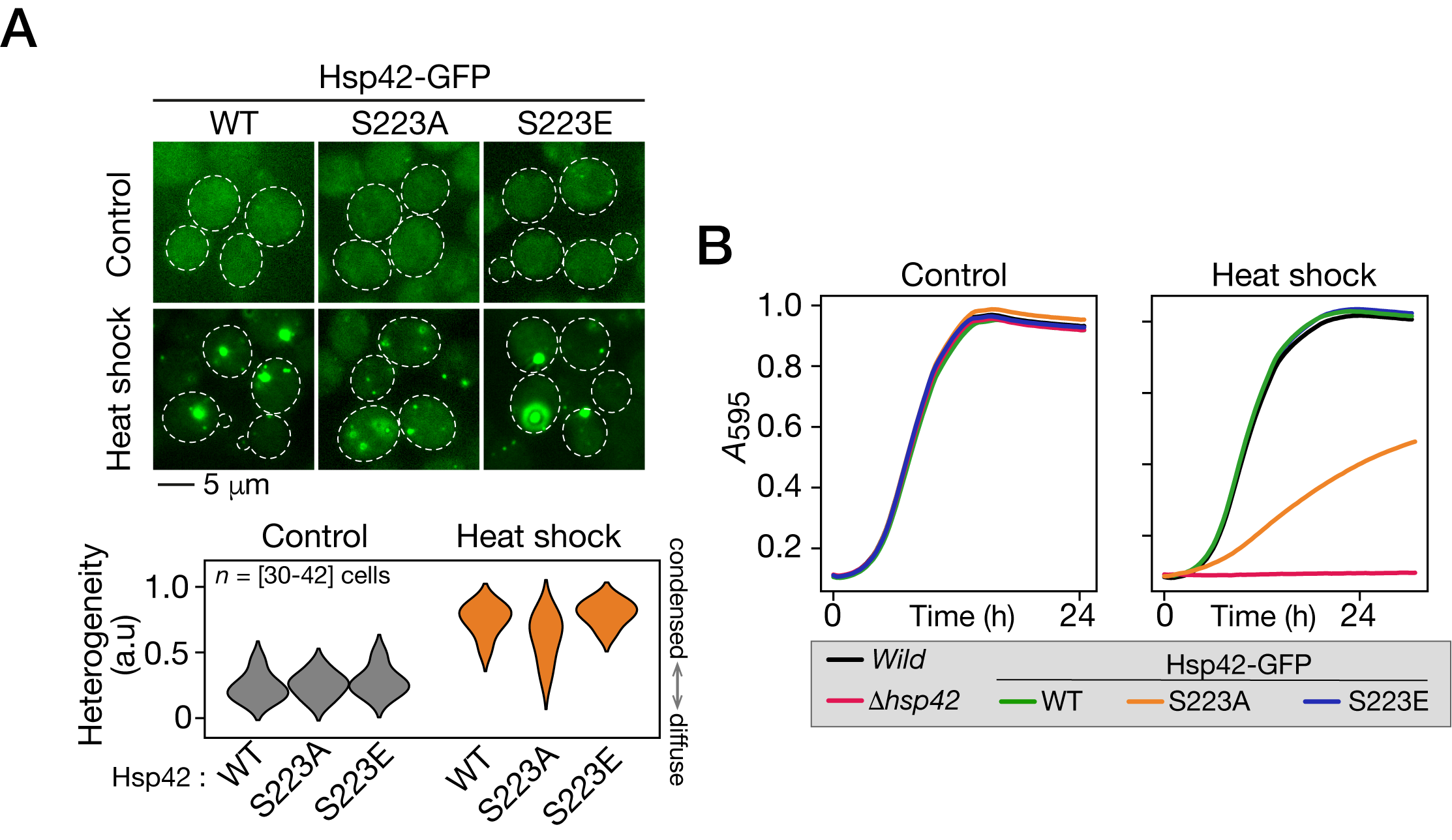

Supplement: S6 Fig — (A) Fluorescence microscopic images of WT or mutant Hsp42-GFP expressing cells, in control conditions (top) or after a heat shock (bottom). Dotted lines represent cell contours. Scale bar represents 5 μm. Bottom, cellular Hsp42-GFP heterogeneity measure. The S223A Hsp42 mutant shows smaller and fainter aggregates in cells, and lower heterogeneity score compared to WT and phosphomimetic mutant. Heat shock at 50°C for 10 min. (B) Growth curves of vegetative cells of the indicated strains after a heat shock (right) or a mock treatment at control temperature (left). Shown are the mean values of 3 replicates. The S223A Hsp42 mutant shows intermediate heat shock resistance between the WT (and S223E mutant) and HSP42 deleted cells. This confirms that the phosphorylation of Hsp42 at this site is important for its function. The data underlying this figure can be found in S6 Data. (TIF) [file pbio.3002042.s006.tif]

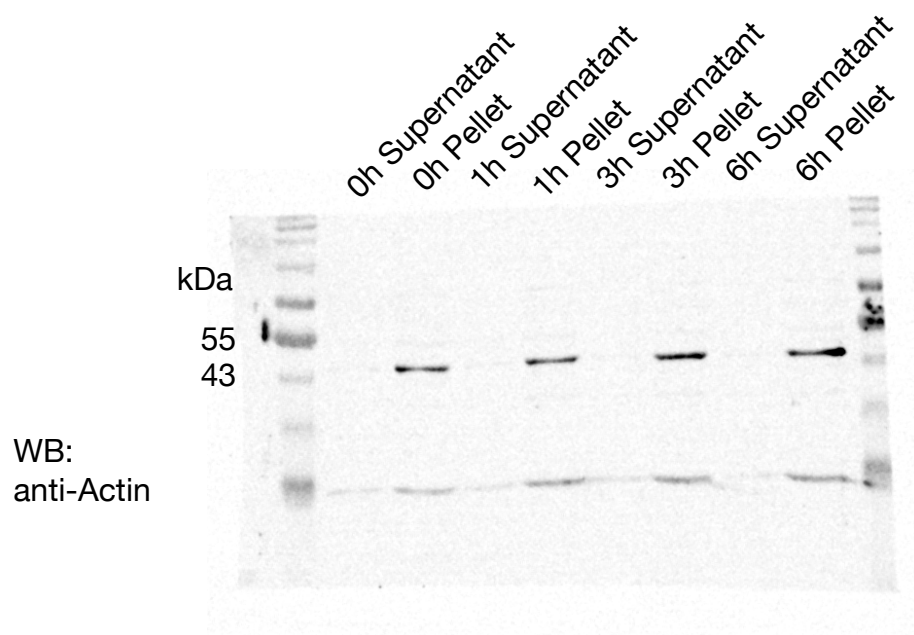

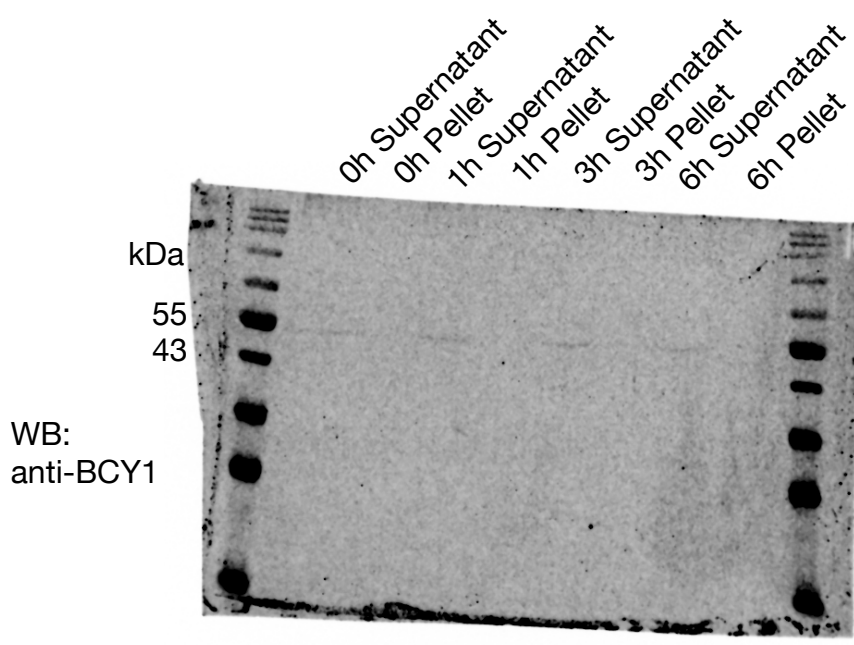

WB:  
anti-Homocitrate  
synthase

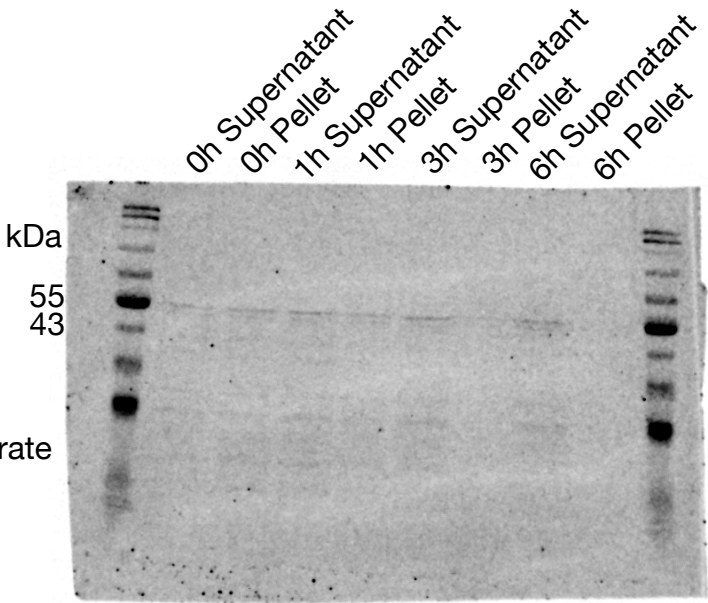

Coomassie Blue  
staining

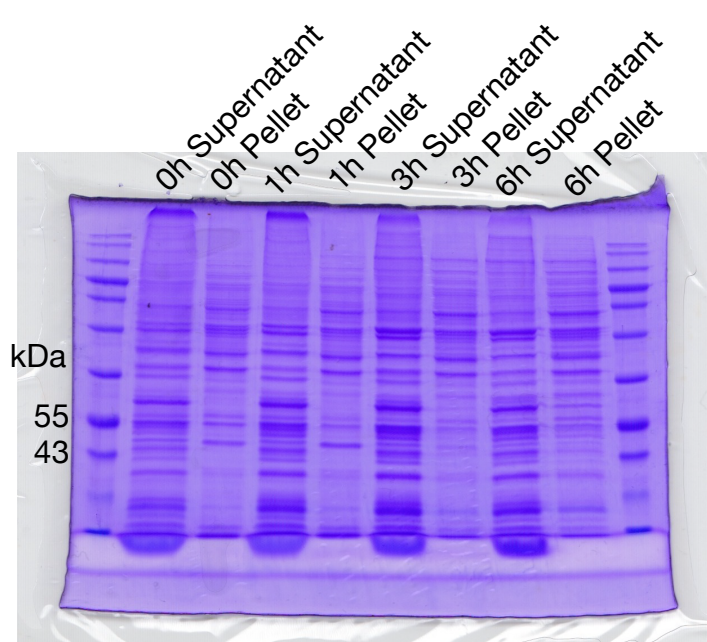

Supplement: S1 Raw Images — (PDF) [file pbio.3002042.s021.pdf]
