# Supplementary material for: Breaking spore dormancy in budding yeast transforms the cytoplasm and the solubility of the proteome
Source: PLoS Biol. 2023 Apr 20;21(4):e3002042. doi: 10.1371/journal.pbio.3002042 (PMC10118125; doi:10.1371/journal.pbio.3002042)
Supplement: S1 Table — (DOCX) [file pbio.3002042.s007.docx]

Key resource table S1

| REAGENT or RESOURCE | SOURCE | IDENTIFIER |
| --- | --- | --- |
| Chemicals, peptides, and recombinant proteins | | |
| cOmplete, EDTA-free Protease Inhibitor Cocktail | MiliporeSigma | cat#11836153001 |
| Percoll | MiliporeSigma | cat#​​P1644 |
| Nigericin | MiliporeSigma | cat#481990 |
| 2-Deoxyglucose | Bioshop | cat#DXG498 |
| Concanavalin A | MiliporeSigma | cat#C2010 |
| Critical commercial assays | | |
| BCA Protein Assay Kit | Novagen | Cat#71285 |
| Deposited data | | |
| Raw and analyzed mass spectrometry data | Data are available via ProteomeXchange | PXD035403 |
| Experimental models: *Saccharomyces cerevisiæ* strains | | |
| LL13_054 wild diploid strain MATa/𝞪 | [[1]](https://paperpile.com/c/lNT1q6/E6zNZ) | LL13_054 |
| ura3::P*SOD1*-µNS-GFP hphNT1  (background: LL13_054) | This Paper | SPY020 |
| ura3::P*SOD1*-sfpHluorin hphNT1  (background: LL13_054) | This Paper | SPY031 |
| ACC1-GFP::hphNT1  (background: LL13_054) | This Paper | SPY037 |
| URA7-GFP::hphNT1  (background: LL13_054) | This Paper | SPY039 |
| HSP42-GFP::hphNT1  background: LL13_054) | This Paper | SPY040 |
| GLK1-GFP::hphNT1  (background: LL13_054) | This Paper | SPY044 |
| hsp42∆::KanMX4  (background: LL13_054) | This Paper | SPY056 |
| hsp42∆::HSP42-GFP-hphNT1  (background: LL13_054) | This Paper | SPY078 |
| hsp42∆::HSP42(S223A)-GFP-hphNT1 (background: LL13_054) | This Paper | SPY080 |
| hsp42∆::HSP42(S223D)-GFP-hphNT1  (background: LL13_054) | This Paper | SPY081 |
| ACC1-mCherry::natNT2  (background: LL13_054) | This Paper | SPY089 |
| hsp42∆::KanMX4 ACC1-mCherry::natNT2  (background: LL13_054) | This Paper | SPY093 |
| hsp42∆::HSP42-GFP-hphNT1 ACC1-mCherry::natNT2  (background: LL13_054) | This Paper | SPY101 |
| hsp42∆::HSP42(S223A)-GFP-hphNT1 ACC1-mCherry::natNT2  (background: LL13_054) | This Paper | SPY102 |
| hsp42∆::HSP42(S223D)-GFP-hphNT1 ACC1-mCherry::natNT2  (background: LL13_054) | This Paper | SPY103 |
| Oligonucleotides | | |
| Primers used in this study are listed in table S1 | This study | N/A |
| Recombinant DNA | | |
| Plasmid: pYM25 | PCR tool box | Janke et al., Yeast, 2004 |
| plasmid: pYM25-PSOD1-µNS-yeGFP | This study | N/A |
| plasmid: pYM25-PSOD1-sfpHluorin | This study | N/A |
| plasmid: pUG6 | Euroscarf | P30114 |
| plasmid: pNATCRE | [[2]](https://paperpile.com/c/lNT1q6/VYf9C) | pNATCRE |
| plasmid: pBS35 (mCherry) + natNT2 | Addgene | Cat#83797 |
| plasmid: pYM25-HSP42-GFP | This study | N/A |
| plasmid: pYM25-HSP42(S223A)-GFP | This study | N/A |
| plasmid: pYM25-HSP42(S223D)-GFP | This study | N/A |
| Software and algorithms | | |
| Rstudio | Rstudio | RRID: SCR_000432 (https://www.rstudio. com/) |
| Python (v 3.7.4) | Python | https://www.python.org/ |
| GrowthCurver (v 0.3.1) | [[3]](https://paperpile.com/c/lNT1q6/mshXR) | https://github.com/sprouffske/growthcurver |
| TrackPy (v 0.5.0) | [[4]](https://paperpile.com/c/lNT1q6/GLqFk) | http://soft-matter.github.io/trackpy/v0.5.0/ |
| Matplotlib (v 3.5.1) | [[5]](https://paperpile.com/c/lNT1q6/BiOh4) | https://matplotlib.org/ |
| Seaborn | [[6]](https://paperpile.com/c/lNT1q6/x5o75) | https://seaborn.pydata.org/index.html |
| Prion-Like Amino Acid Composition (PLAAC) | [[7]](https://paperpile.com/c/lNT1q6/KGHrI) | http://plaac.wi.mit.edu |
| scipy.cluster.hierarchy (v1.8.1) | Scipy | https://docs.scipy.org/doc/scipy/reference/cluster.hierarchy.html |
| Metapredict (v2.0) | [[8]](https://paperpile.com/c/lNT1q6/a8VdG) | https://github.com/idptools/metapredict |
| Phase Separation Analysis and Prediction (PSAP) | [[9]](https://paperpile.com/c/lNT1q6/UOZTR) | https://github.com/Guido497/phase-separation). |
| Scikit-learn (sklearn) v1.1.1 | [[10]](https://paperpile.com/c/lNT1q6/nQJZ8) | https://scikit-learn.org/ |

References

1. [Leducq J-B, Nielly-Thibault L, Charron G, Eberlein C, Verta J-P, Samani P, et al. “Speciation driven by hybridization and chromosomal plasticity in a wild yeast.” Nat Microbiol. 2016;1: 15003.](http://paperpile.com/b/lNT1q6/E6zNZ)

2. [Steensma HY, Ter Linde JJ. “Plasmids with the Cre-recombinase and the dominant nat marker, suitable for use in prototrophic strains of Saccharomyces cerevisiae and Kluyveromyces lactis.” Yeast. 2001;18: 469–472.](http://paperpile.com/b/lNT1q6/VYf9C)

3. [Sprouffske K, Wagner A. “Growthcurver: an R package for obtaining interpretable metrics from microbial growth curves.” BMC Bioinformatics. 2016;17: 172.](http://paperpile.com/b/lNT1q6/mshXR)

4. [Crocker JC, Grier DG. “Methods of Digital Video Microscopy for Colloidal Studies.” J Colloid Interface Sci. 1996;179: 298–310.](http://paperpile.com/b/lNT1q6/GLqFk)

5. [Hunter JD. “Matplotlib: A 2D Graphics Environment.” Computing in Science & Engineering. 2007;9: 90–95.](http://paperpile.com/b/lNT1q6/BiOh4)

6. [Waskom ML. “Seaborn: statistical data visualization.” Journal of Open Source Software. 2021;6: 3021.](http://paperpile.com/b/lNT1q6/x5o75)

7. [Lancaster AK, Nutter-Upham A, Lindquist S, King OD. “PLAAC: a web and command-line application to identify proteins with prion-like amino acid composition.” Bioinformatics. 2014;30: 2501–2502.](http://paperpile.com/b/lNT1q6/KGHrI)

8. [Emenecker RJ, Griffith D, Holehouse AS. “Metapredict: a fast, accurate, and easy-to-use predictor of consensus disorder and structure.” Biophys J. 2021;120: 4312–4319.](http://paperpile.com/b/lNT1q6/a8VdG)

9. [van Mierlo G, Jansen JRG, Wang J, Poser I, van Heeringen SJ, Vermeulen M. “Predicting protein condensate formation using machine learning.” Cell Rep. 2021;34: 108705.](http://paperpile.com/b/lNT1q6/UOZTR)

10. [Pedregosa F, Varoquaux G, Gramfort A, Michel V, Thirion B, Grisel O, et al. “Scikit-learn: machine learning in python.” JMLR 12: 2825--2830. J Mach Learn Res. 2011;12: 2825–2830.](http://paperpile.com/b/lNT1q6/nQJZ8)
