# Supplementary material for: Breaking spore dormancy in budding yeast transforms the cytoplasm and the solubility of the proteome
Source: PLoS Biol. 2023 Apr 20;21(4):e3002042. doi: 10.1371/journal.pbio.3002042 (PMC10118125; doi:10.1371/journal.pbio.3002042)
Supplement: Attachment — Submitted filename: Response to reviewers.pdf [file pbio.3002042.s022.pdf]

December 19, 2022

Dr Roland Roberts, PhD
Senior Editor, PLOS Biology

Dear Dr Roberts,

We are pleased that our manuscript received a broadly positive review. We are thankful to the reviewers for their thorough and helpful comments. We made numerous changes to the text and figures and performed additional experiments to satisfy their requests. We are confident that our paper will now make a stronger contribution to PLOS Biology.

You will find our detailed response below. Our responses are in blue and each time, we reproduce the section that was modified (yellow highlighted text).

Looking forward to hearing from you.

Christian

Christian R. Landry
Université Laval
1030, avenue de la Médecine
Québec (Québec)
Canada, G1V 0A6

Review 1:

"Throughout the paper: I agree with the authors that dormant spores are highly stress resistant. Therefore, some of the compounds the yeast cells accumulate when stressed to become more stress resistant are likely also present in high concentrations in dormant spores. There is an overlap between dormancy and stress responses, namely both cell types accumulate compounds to stabilize proteins and membranes, therefore allowing the cell to become metabolically (almost) inactive in addition to becoming stress resistant. However, this overlap does not per definition mean that a cell entering dormancy is identical to a cell responding to a stressor. Thus, although I do value the comparison, I feel the authors should be careful when calling a dormant spore a "stress response state" and germlings an "unstressed state". Instead, I would suggest altering the statements slightly, saying that the dormant state has many similarities with a stress response state."

To emphasize that dormant spores are comparable to a stress resistant state, we changed our statements to say that spores share similarities with stress response in yeast, and that germination mimics the stress relief experienced in yeast.

Examples of text that was changed:
“The reverse order of events between spore germination and heat stress and nutrient stress responses for some key proteins suggests a model in which dormancy in spores is analogous to a stress response state and germination corresponds to the relief of the stress state allowing return to metabolic activity and vegetative growth (Figure 3D).”

“-Page 3: In the 2nd Alinea of the introduction, the authors introduce fungal spores as stress resistant cell types of fungi in general. When discussing all fungal spores in a broad sense, I assume this includes conidia, basidiospores and other fungal spore types. However, all information given is solely focusing on yeast ascospores. I would have liked to see some information regarding other fungal spore types, like conidia and basidiospores, or otherwise a clear transition towards the focus on specifically yeast spores.”

Following the sentence “Fungal life cycles include the production of spores” in the introduction, we added the following text to broadly introduce spores and then transition to talk specifically about ascospores:

Although being formed through largely different mechanisms, conidia (asexual spores), and ascospores and basidiospores (sexual spores) have in common to be stable dormant cell types (Dijksterhuis 2019; Wyatt et al. 2013). These cells all show a variety of resistance to extreme conditions such as heat, desiccation (Ho and Miller 1978; Oneto et al. 2020), and many harsh complex environment such as insect guts (Coluccio et al., 2008) or immune system assaults (Levitz and Diamond 1985; Botts and Hull 2010). Because of the increased resistance of spores to extreme conditions, sporulation is thought to be an adaptive strategy to survive changing environmental conditions (Huang and Hull, 2017). In ascospores, which are produced by our model the budding yeast S. cerevisiae, stress resistance is largely attributed to the thick cell wall of specific composition (Neiman, 2011)

“-Page 6: Here the authors investigated the internal pH levels of ascospores by quantifying the fluorescence of a pH sensitive GFP fluorophore called pHluorin. However, I have some concern regarding this methodology. As I recall, the pH sensitivity of this fluorophore is due to conformational changes of the fluorophore during pH changes (this is what "senses" the shift in pH). Is it possible that the pH sensitivity of this fluorophore is altered in a highly viscous environment? Are the conformational changes due to pH shifts still possible when the fluorophore is in this highly stress resistant cell type, where there are high concentrations of osmolytes and a high number of chaperones present, like trehalose and heat shock proteins, that stabilize the protein? It would be beneficial to the manuscript

if the proper functioning of the protein in a highly viscous environment is experimentally tested, or if this has been confirmed before, referred to the paper where this has been shown."

We agree that spore cytosol is an extreme environment, in which we show solubility of many proteins is altered, therefore raising concern about the state in which pHluorin is. To our knowledge, no previous work addressed the question of pHluorin functioning in a viscous environment. To answer this question, we performed a calibration curve on pHluorin expressed in dormant spores. Difference in response between pHluorin in spores and in vegetative yeast would inform us about the effect of viscosity on its sensitivity. We showed this analysis in Figure S1D. Our results revealed that viscosity in spores had only a mild effect on pHluorin sensitivity.

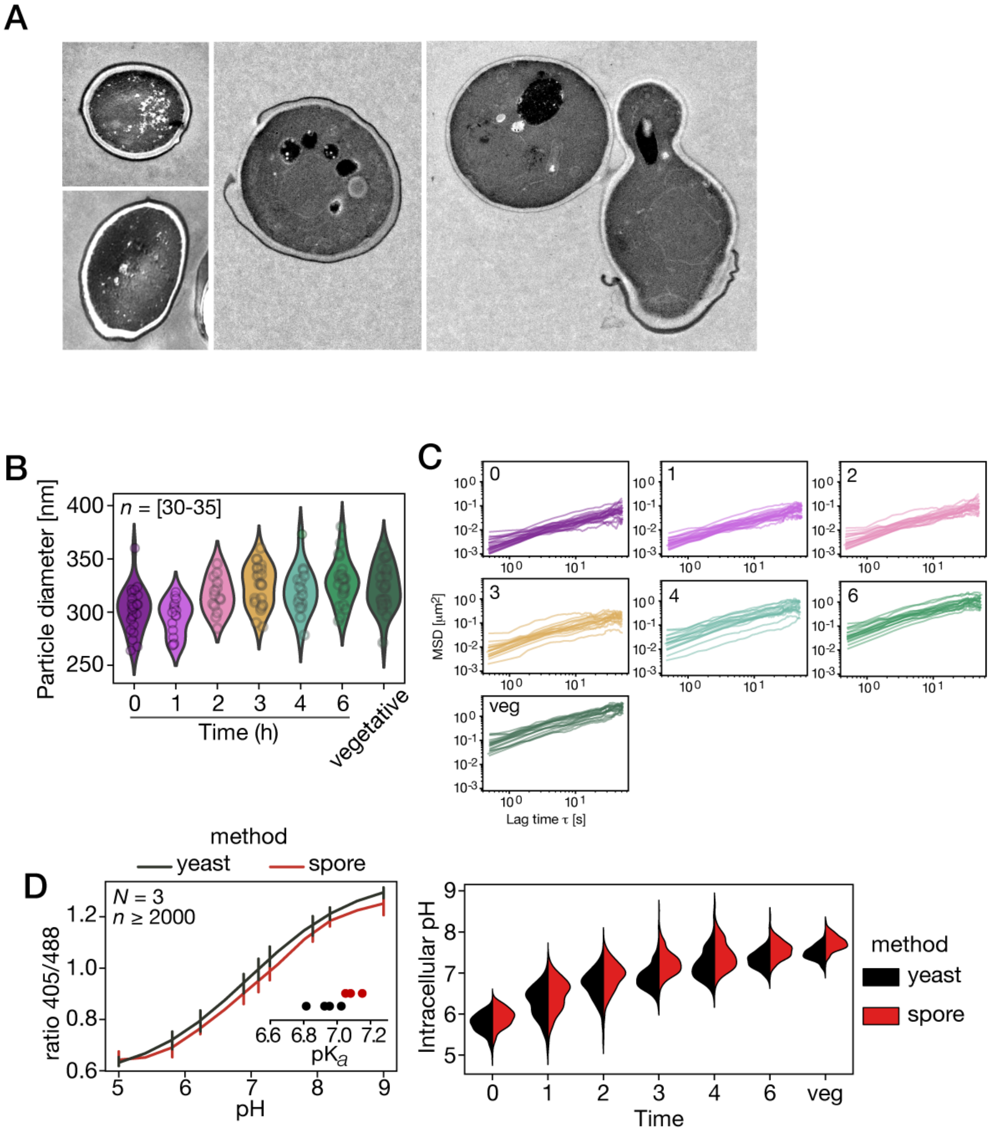

Figure S1 - µNS-GFP particle size and Mean square displacement (MSD) during germination; calibration curve of pHluorin emission ratio, related to figure 1.

A) Transmission electron microscopy images of spores at the indicated time after exposure to rich medium. Scale bar represents 1 µm.
B) Size of individual particles tracked at each time point during germination and in vegetatively growing cells. At least 30 particles were tracked at each time point.
C) Mean square displacement of individual particles tracked at the indicated time after germination induction.
D) Left, intracellular pH calibration curves determined using vegetative yeast (black) or spores (red). Although the curve using spores is slightly more basic, measurement of pH in germination (right) using either curve shows that spores are acidic and that intracellular pH increases steadily during germination. Logistic function fitted to the data from vegetative cells was used for Figure 1G. Error bars represent standard deviation of three replicates.

In addition, we added the underlined text in method section (Subsection "Intracellular pH measurements") to describe the deeping analysis we made of pHluorin function:

Viscosity is among many parameters that affect the response of pHluorin to its environment (Fricker et al. 2001). To confirm that pHluorin sensitivity and response in the highly dense and rigid ascospores cytosol is comparable to that in yeast cells, we performed a calibration curve in spores. Spores expressing pHluorin were incubated in a calibration buffer adjusted to various pH for 1 hour at room temperature before fluorescence was analyzed by flow cytometry as for vegetative yeast. Fluorescence ratios showed that pHluorin in spores have a similar sensitivity and response compared to that in yeast cells (Figure S1D).

"-Page 11: The authors show that germination is delayed in hsp42Δ cells and additionally, that there is a delayed decrease in viscosity during germination of hsp42Δ cells. There have been reports that deletions of certain heat shock proteins that act as chaperones, like Hsp104, is compensated for by the cell, by accumulating more trehalose (Elliott et al. 1996). This compensation has also been suggested for Hsp42 (Haslbeck et al. 2004), although I am not aware if this relation with Hsp42 specifically has been proven. My question: is it possible that your hsp42Δ cells accumulate more trehalose than wild-type cells and that this increased concentration of trehalose causes this delayed decrease in viscosity during germination? The potential change in internal compatible solute concentrations of these hsp42Δ cells could perhaps also influence other aspects of the conclusions. Is Hsp42 a key actor for the solubilization of low solubility proteins, or could some of these findings be explained by a change in internal compatible solute concentrations? It would strengthen the paper significantly if compatible solute concentrations, or at least trehalose concentrations, are determined for these strains during germination, or otherwise if the potential role of compatible solutes is discussed in the manuscript (see next remark)."

The reviewer raised an excellent question. To answer the question whether trehalose might accumulate at higher levels in Δhsp42 spores, and explaining the germination delay we observed in these spores, we measured trehalose content during germination. We saw that trehalose content in Δhsp42 spores remained high after germination induction, while its level dropped quickly in WT spores. Our results were included in Figure 5 as follows.

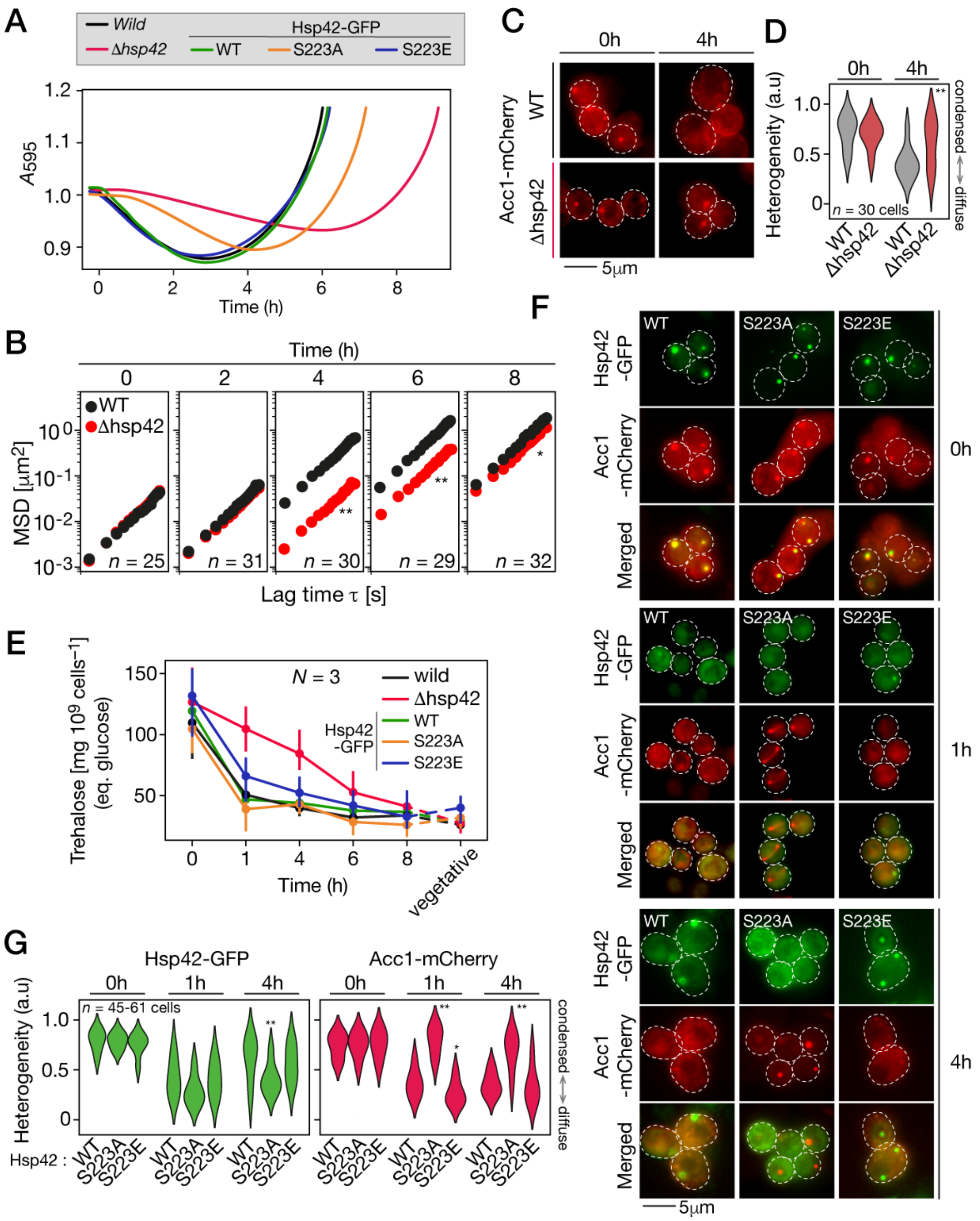

Figure 5 - Active and phosphorylated Hsp42 is required for normal germination dynamics.

A) Optical density of pure spore cultures of the indicated strains as a function of time following exposure to germination conditions. Shown are the mean values of three replicates. OD drop of the Δhsp42 spores is strongly delayed, indicating that germination is inhibited or slowed down.

Spores expressing S223A Hsp42 mutant show a slight delay in germination, while germination of spores expressing S223E Hsp42 mutant is indistinguishable from that of wild spores.
B) Ensemble mean square displacement of μNS-GFP in WT and Δhsp42 spores at the indicated time after exposure to rich medium. At each time point for each strain, 25 to 35 particles, corresponding to the same number of cells, were tracked. Kruskal-Wallis test, ** indicates p-value < 0.0001, * indicates p-value < 0.01.
C) Fluorescence microscopy images of wild type (top) or ΔHsp42 spores expressing Acc1-mCherry at the indicated time after exposure to germination conditions. Scale bar represents 5μm.
D) Cellular Acc1-mCherry heterogeneity score in WT or Δhsp42 spores at the indicated time after exposure to rich medium.
E) Trehalose content (measured in equivalent of glucose concentration) in spores at the indicated time after exposure to rich medium, and in vegetative yeasts. Error bars represent standard deviation of three replicates.
F) Fluorescence microscopy images of spores expressing either WT, S223A or S223E mutant Hsp42-GFP and Acc1-mCherry at the indicated time after exposure to germination conditions. Dotted lines represent cell contour. Scale bar represents 5 μm. Expression of S223A Hsp42-GFP mutant shows delay in Acc1 foci dissolution in spores.

"-Page 12: One important topic I feel is lacking in the manuscript, and specifically in the discussion, is the potential role of trehalose. Trehalose accumulates in high concentration in fungal spores and is potentially one of the (if not main) reasons that the spores become more viscous. Trehalose increase is also a known stress response, and therefore would perfectly fit the comparison the authors make between dormancy and stress response state. Can the authors include some relevant information on the role of trehalose in the discussion, and reflect on the potential role of trehalose in dormancy breaking in comparison to Hsp42?"

In addition to the trehalose content determination (Figure 5E, see last point) we added the following text in the Results & Discussion section to discuss the role of trehalose and its relation with Hsp42.

Yeast adaptation to various stressor includes accumulation of compatible solute, notably the disaccharide trehalose (D'Amore et al. 1991). It promotes survival by stabilizing macromolecules such as membranes and proteins (Kaushik and Bhat 2003; Magalhães et al. 2018). Fungal spores, including *S. cerevisiæ* ascospores, accumulate trehalose which account as a protective feature they develop (Kane and Roth 1974). Accumulation of trehalose was found to increase yeast cytoplasm viscosity as a homeostatic mechanism to maintain molecule diffusion rate in response to stress and energy depletion (Persson et al. 2020). Mobilization of trehalose is essential for yeast spore germination since inhibition of trehalase activity in spores hinder germination (Thevelein et al. 1984; Beltran et al. 2000). Here, we investigated trehalose content in the course of germination to see if it could play a role in the changes we measured. Both wild type and *hsp42*Δ spores accumulated a high level of trehalose. In wild type spores, trehalose content dropped by >55% after one hour in germination medium (Figure 5E), which is in line with previous works reporting a quick mobilization of trehalose content in the first hour of

germination (Rousseau et al. 1972; Thevelein et al. 1982). Contrastingly, trehalose content in hsp42Δ spores remained higher than the level in WT spores for the first four hours of germination, and then decreased down to WT level after 8 hours (Figure 5E). The delay in trehalose mobilization in *hsp42Δ* spore suggest that Hsp42 contributes to germination upstream of trehalose mobilization and that the major changes observed in the cytoplasm of *hsp42Δ* cells are at least partly caused by the dynamics of trehalose mobilization.

Also, follow the sentence "Hsp42 presumably functions in adapting spore cytosol to nutrient repletion in a similar manner as Floe1 integrates the signal of adequate hydration in A. thaliana seed to control germination (Dorone et al., 2021)" in the conclusion section, we added the text: The first event involving Hsp42 is its solubilization occuring at the breaking of dormancy. The timing of Hsp42 foci dissolution at the onset of breaking of dormancy indicated that it is likely triggered by the pathways initiating germination. While this hypothesis is not substantiated in this work, it will be worth considering the mobilization of trehalose, which functions as a macromolecule stabilizer in dormant spores, as a factor that could interact with Hsp42 to modulate the solubility of the cytoplasm.

"-page 13: "across the three of life" > "across the tree of life"
We corrected this mistake.

Reviewer 2:

"1. Quantification and causality. I was struck by the differences between the kinetics of the of the uNS vs. pHluorin measurements during germination in 1D vs 1F, and how these are both much slower than the complete loss of heat resistance. This made we wonder about causality. Can the authors do an experiment that artificially perturbs pH and/or viscosity? Their argument would be greatly strengthened by experiments that move us beyond correlative understanding. This approach would also help the authors to include a control that appears to be missing for the robustness of the pHluorin measurements to changes in cytoplasmic viscosity."

We agree that differences between kinetics of pH and fluidity measurement are puzzling. Previous works that inspired us for these measurements showed that in energy depleted yeast, shifts in intracellular pH resulted in quick variation in cytoplasm viscosity. We believe that our results reveal that in the context of spore germination, possibly many more factors contribute to cytoplasm fluidity changes. Following the sentence "At this point, intracellular pH gets close to that measured in vegetatively growing cells (pH_i = 7.4)" in the results section, we added the underlined text to discuss the relation between pH and viscosity/fluidity of the cytoplasm: Previous works showed that acidification and alkylation of yeast cytosol causes reduction and increase of motility of μNS particles respectively, and that this effect happens quickly, in the scale of a few minutes (Munder et al. 2016). However, our results show that during germination, the kinetics of change in particle mobility is delayed compared to change of intracellular pH.

Although germination involves physicochemical changes related to that seen in vegetative yeast recovering from stress, they are modulated in a germination specific manner. Our results reveal the contribution of possibly many other factors to changes in viscosity.

"2. What does the centrifugation procedure capture, and what does it exclude? I would appreciate a fuller discussion of exactly what particle sizes are captured in the centrifugation, and what is not. Following some marker proteins by immunoblot would be especially useful. I also think cell wall/membrane digestion experiments, as have been done by Simon Alberti's group with S. pombe, would be very helpful as an orthogonal approach to distinguish between the viscous model proposed and others that involve bona fide solids."

We agree that a more comprehensive characterization of the separation process is needed. In order to grasp the extent of physical separation we executed, we performed a GO term analysis on the clusters specifically regarding cellular component terms. Different cell components are enriched in the different clusters, showing that our separation technique performed well. Moreover, using available antibodies against endogenous yeast proteins, we corroborated by western blot analysis the Pindex profile of few proteins. We included this analysis in Figure 2C, and to respond to the comment, we presented the results as a bubble plot:

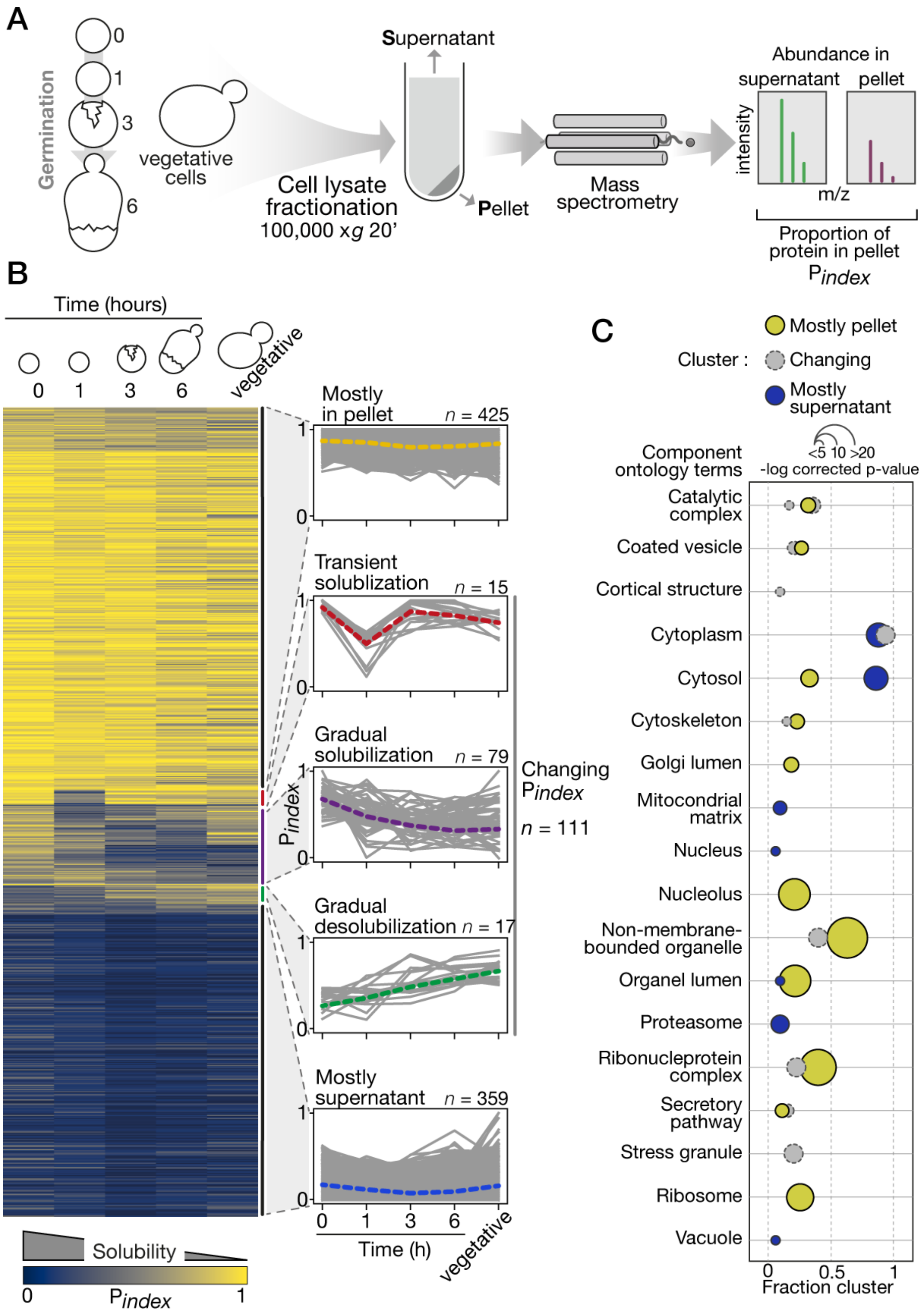

Figure 2 - Proteome wide change in protein solubility during germination.
A) Solubility measurement by LC-MS/MS estimates the proportion of each protein in the pellet (P_{index}) at each major time-point sampled during germination. The experiment was performed in triplicate for all timepoints.

B) Right, P_{index} values in the course of germination show, from top to bottom, proteins consistently found in the pellet, that transiently solubilize, that gradually solubilize, that gradually accumulate in the pellet, and that are consistently found in the supernatant. Left, individual P_{index} trajectories for each cluster determined by hierarchical clustering. The dotted line is the median trajectory for each cluster.
C) Gene ontology term analysis focused on cellular component terms. Terms enriched for each cluster (Mostly in pellet, Changing Pindex and mostly supernatant) are shown as bubble plots. Colors refer to the cluster, position on the x-axis indicates the portion of the proteins in a cluster assigned to a GO term, and size of the bubble is scaled to the -log (*p*-values). See Figure S2 for additional details.

We performed the western blot analysis on **actin** (remains in pellet), **homocitrate synthase** (Lys20/21, gradually solubilizes) and **Bcy1** (remains in supernatant). We included the results in Figure S3.

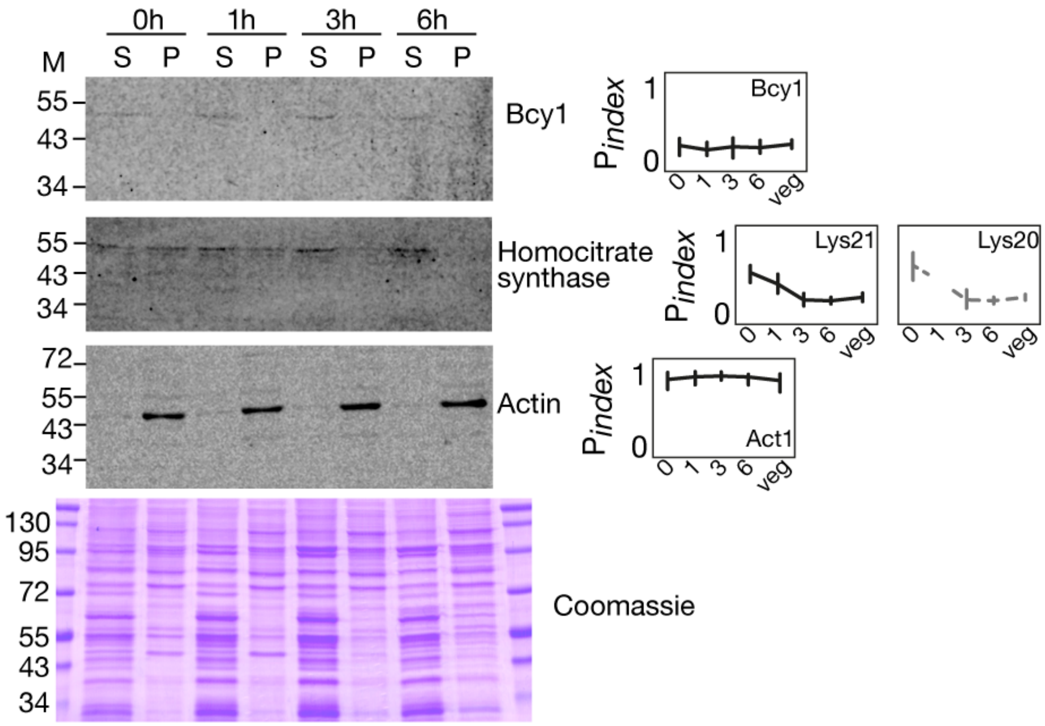

Figure S3 - Protein sedimentation reveals different protein solubility, related to Figure 2
Left, Same fractionned protein extract (S, supernatant fraction; P, pellet fraction) used in MS measurements were analyzed by SDS-PAGE with antibodies that were available for yeast endogenous proteins. Shown are anti-Bcy1, anti-Homocitrate synthase and anti-actin western blots, and an identically loaded gel stained by Coomassie. Proteins molecular weight in the ladder (NEB# P7706) are indicated in KDa.
Right, P*index* trajectories of the proteins analyzed by western blot. Homocitrate synthase isozyme Lys20 was poorly detected at 1 hour time-point. Error bars represent standard deviation of three replicates.

We discussed these new analyses in the text.

Following the sentence “Finally, 79 proteins with varying single trajectories gradually gained solubility during germination” in the results section, we added:

Gene ontology analysis, using the total protein considered for our analysis as a reference set, revealed that clusters are enriched for different cell component terms (Figure 2C). While the mostly supernatant cluster appears to contain essentially cytosolic proteins, the proteins in the mostly pellet and changing Pindex clusters are assigned to various and more specific cellular components. For instance, the later clusters are both enriched for proteins in non-membrane-bounded-organelles, ribonucleoprotein complexes and cytoskeleton. There is a specific enrichment for nucleolar and ribosomal protein in the mostly pellet cluster, and a specific enrichment for stress granule proteins in the changing Pindex cluster. These results highlight the level of separation performed by our technique, which seems to separate non-membrane-bounded organelles and macromolecular complexes from the other constituents of the cytosol.

And following “ Replicated measurements were strongly correlated (Figure S2A)” we added the text:

Moreover, the solubility profile of endogenous proteins in the fractionned cell extracts revealed by western blot is similar to their Pindex trajectories (Figure S3).

“3. Including additional data in the figures. The figures are beautiful but very minimalistic. A couple of suggestions to increase information content: a) in Fig. 3A consider showing a fuller GO term analysis. Maybe as a heatmap or bubble plot. KEGG may also be useful in this respect.”

As suggested, we changed Figure 3 with a bubble plot. The graph shows the complete set of GO terms found.

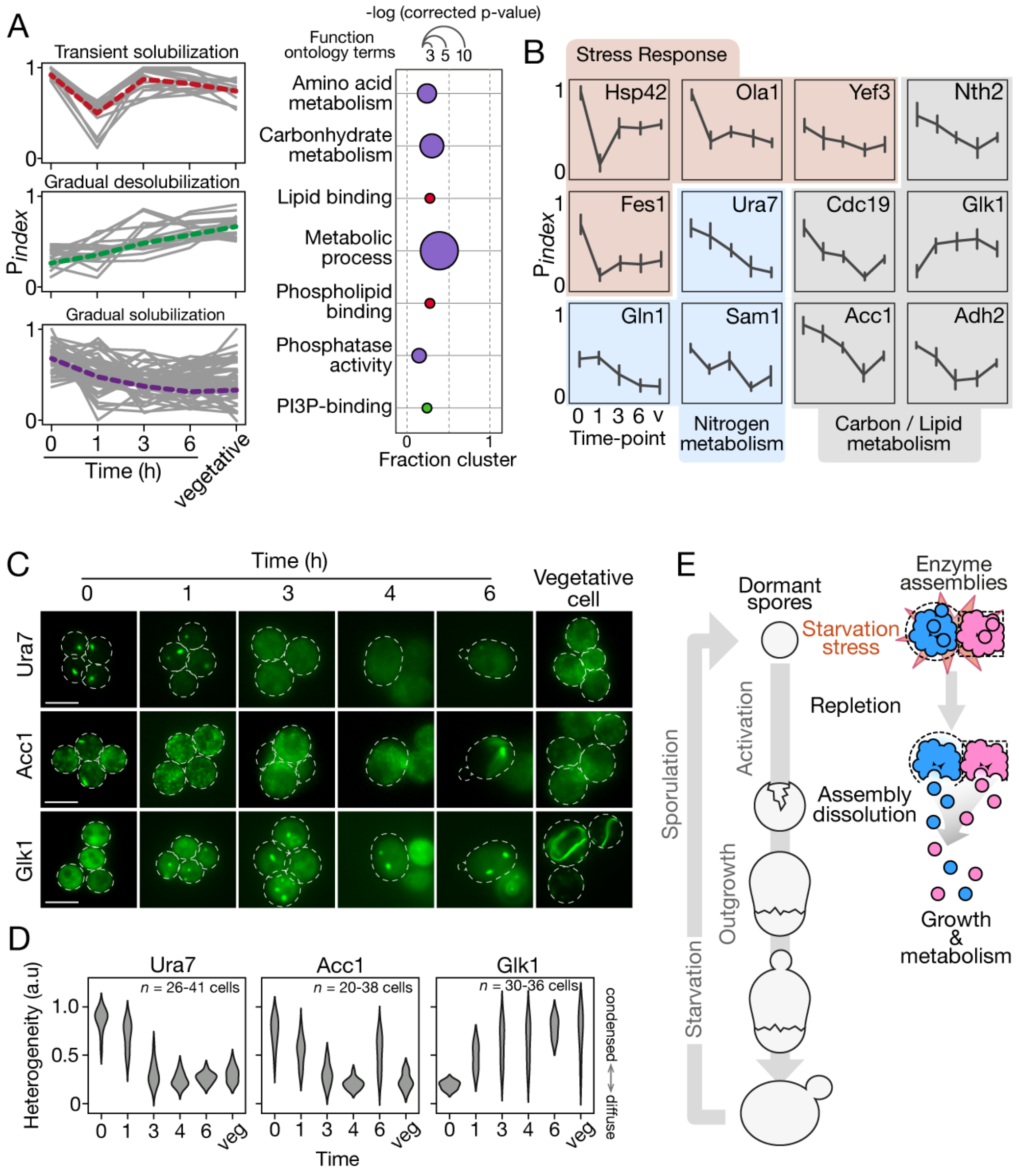

Figure 3 - Solubility changes reflect metabolism activation and mimic stress relief during germination.

A) Enrichment for gene ontology terms in each dynamically changing solubility cluster. Red, transiently solubilizing cluster; Green, gradual desolubilization cluster; Purple, gradual solubilization cluster. The position on the x-axis indicates the portion of the proteins in a cluster assigned to a GO term, and size of the bubble is scaled to the -log (*p*-values) from a hypergeometric test.

B) Individual P_{index} trajectory for representative proteins through germination. Bar represents standard variation. Proteins are clustered by function; Red, stress response proteins; Blue, Nitrogen metabolism proteins; Gray, Lipid and carbon metabolism proteins. Error bars represent standard deviation of three replicates.

C) Representative fluorescence microscopic images of spores expressing the indicated proteins tagged with GFP during germination. Top to bottom, Acetyl-CoA carboxylase Acc1 (Lipid

biosynthesis), CTP synthase Ura7 (pyrimidines synthesis) and Glucokinase Glk1 (glycolysis). The Glk1 foci formation and the dissolution of Acc1 and Ura7 foci in course of germination support that dormancy in spores is analogous to a stress state and germination alleviates this state. Dotted lines indicate cell contour determined by brightfield images. Scale bars represent 5µm.
D) Measure of cellular heterogeneity (coefficient of variation) of the fluorescent proteins in spore at the indicated time after exposure to rich medium or in vegetative cells. Between 20 and 41 cells were analyzed at each time-points.
E) Schematics highlighting effects on protein solubility of nutrient starvation and repletion during sporulation and germination, respectively. Pink and blue assemblies represent assemblies of enzymes needed for growth and metabolism during dormancy, which disassemble (pink and blue circles) during germination.

"b) in Fig. 4A please provide the common names of the proteins."

As suggested, we added the common names of the proteins in Figure 4A

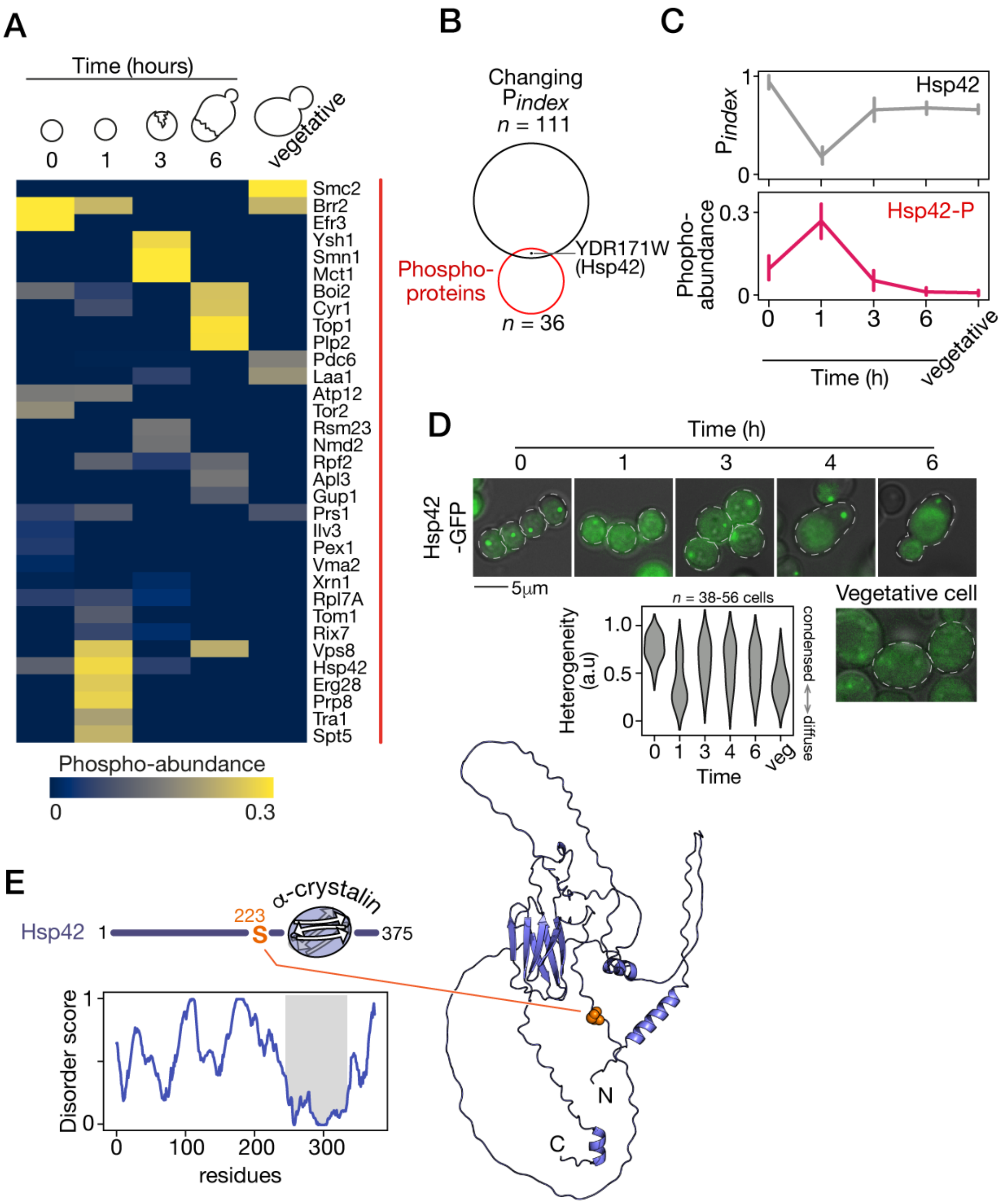

Figure 4 - Hsp42 phosphorylation at S223 is synchronized with its transient solubilization.

A) Relative abundance of the 36 phosphoproteins to the total abundance of each protein through germination.

B) Hsp42 is phosphorylated during germination and changes solubility. See Figure S3 for additional information.

C) Hsp42 is the only protein with dynamic solubility profile during germination that correlates with its dynamic phosphorylation, here at S223. Error bars represent standard deviation of three replicates.

D) Representative fluorescence microscopic images of spores expressing Hsp42-GFP at the indicated time after the induction of germination. Dotted lines represent cell contour. Scale bar

represents 5µm. Plot shows the cellular Hsp42-GFP heterogeneity score in spore at the indicated time after exposure to rich medium or in vegetative cells.
E) Left, Disorder profile of Hsp42, predicted by Metapredict, shows the predicted structured ACD domain, and flanking disordered N- and C-terminal region. Right, predictedHsp42 structure. The S223 highlighted in orange is located in a disordered region.

c) in Fig. 3B how do the trajectories for other representative chaperone proteins compare? I would suggest showing - in the main probably but if not certainly in the supplement - the identities and traces for all (n=15) proteins with a similar solubilization profiled to Hsp42.

We included in Figure S5 the trajectories and identities of the other proteins in the Transient solubilization cluster. Also, we included the trajectories of the other molecular chaperones (Hsp104, Hsp26, Hsp82, Ssa1, Ssa2)

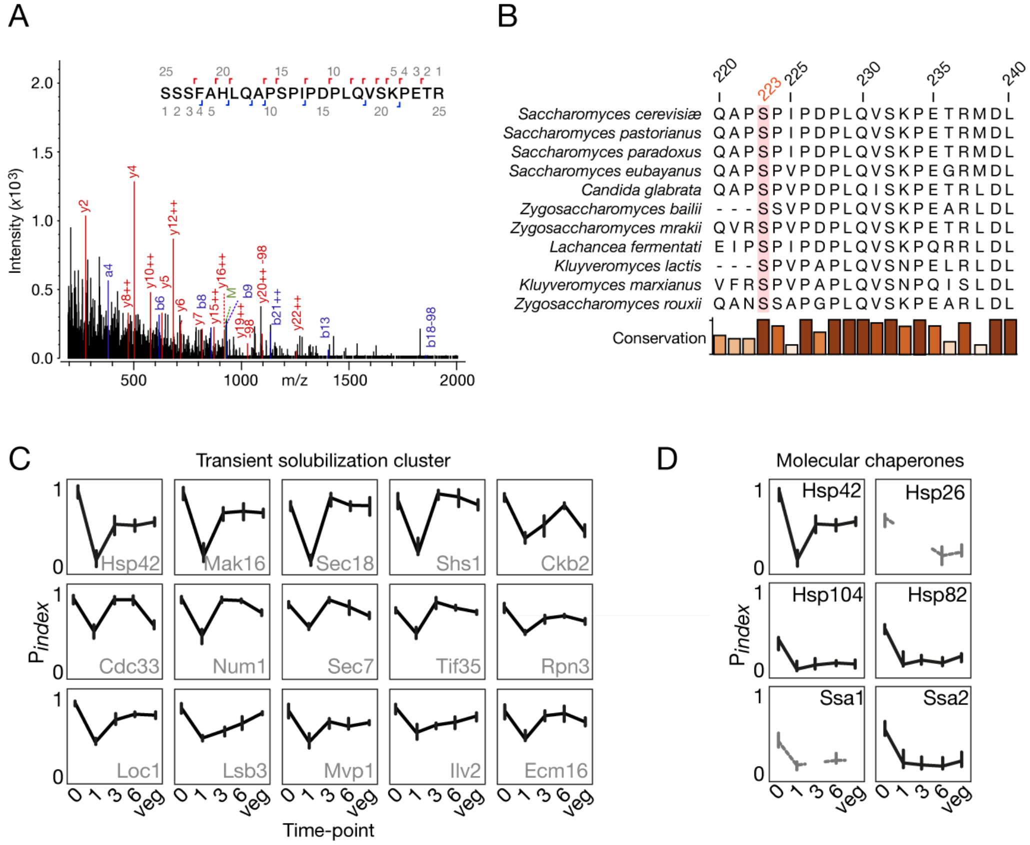

Figure S5 - Phosphorylation of Hsp42 on S223, Transient solubilization cluster and molecular chaperones, related to Figure 4.
A) MS spectra example of phosphorylated S223 peptide on Hsp42.
B) Multiple Sequence Alignment of Hsp42 orthologs. Numbers on top refer to residue position in the *S. cerevisiae* protein. S223 is underlined in orange. Relative conservation is shown with the bars at the bottom. Only a small portion of the sequences are shown.
C) Individual P*index* trajectories for each 15 proteins in the transient solubilization cluster. Error bars represent standard deviation of three replicates.

D) P*index* trajectories of molecular chaperones detected in our experiments. Hsp26 and Ssa1 have only partial data since they are not well detected. However, these data reveal that Hsp42 have a unique sedimentation profile among molecular chaperones. Error bars represent standard deviation of three replicates.

"4. Is the solubility change in 111 proteins due to a couple of condensates dissolving, or changes in the bulk materials properties of the cytoplasm? I would recommend some more extensive comparisons of the 111 proteins found here to those identified in other studies in yeast. Stress granules of course, but also Q bodies and other structures such as the APOD, which is notable for its sequestration of Hsp42. More broadly, I would suggest that the authors investigate how much of what they are seeing is due to general cytoplasmic properties as opposed to a specific condensate composed of Hsp42, Acc1, and perhaps many of the other proteins? One gets some suggestions that there may be more than one assembly from Fig. 3C, but I would be more convinced by additional examples and analyses to investigate this point. Even something as simple as an analysis of which of the 111 proteins with changed solubility interact with one another in systematic P-P interaction data would be very useful."

This comment raised an excellent question about the relation of the 111 proteins we found changing Pindex during germination. Through GO term analysis (see Figure 2C) we found that proteins in the changing Pindex group are enriched for non-membrane bounded organelle, ribonucleotide complexes and stress granules. This result reveals that these proteins are prompte to condensation. To understand whether they are linked to common clusters or condensates, we searched for known protein-protein interaction among the 111 changing Pindex proteins. We found only 6 pairs of interacting proteins. The interacting partners we identified are not involved in known condensate formation, which suggests that foci dissolution we observed does not reflect changes in specific interaction. We showed these results in Figure S4.

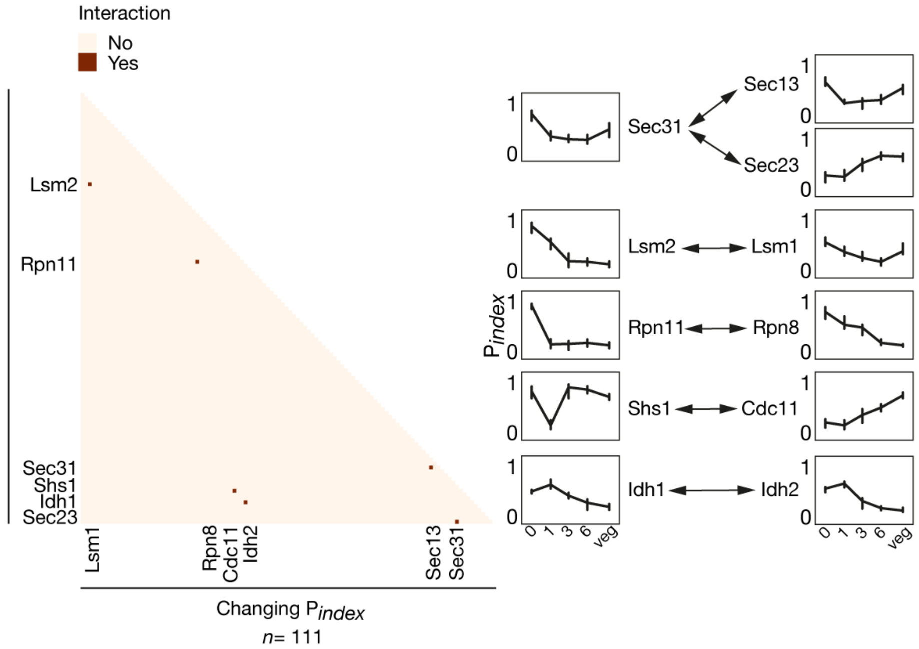

Figure S4 - Known protein-protein interaction among proteins of the changing Pindex group.

Left, interaction between the 111 Changing Pindex proteins were searched through the known physical interaction database (BioGRID v4.4.216). Pairs of interacting proteins are marked in red in the heat map with the identity of the partners on the side. Right, the 6 pairs of interacting proteins are indicated with arrows, and the Pindex trajectories for each protein is shown. Error bars represent standard deviation of three replicates.

We also included the underlined text to discuss this result. :

In addition, the analysis of known physical interaction among the detected proteins revealed that changes we reported in P*index* do correlate with large interaction networks. Out of the 111 changing Pindex proteins, 6 pairs of physically interacting proteins were found (Figure S4). This suggests that the changes in protein organization we observed likely reflect bulk changes in cytoplasm properties rather than remodeling of specific interactions.

"5. Quantification. Quantification and statistical tests are essential for all micrographs. Likewise, error needs to be conveyed for the mass spec data. Just a couple of examples, but this applies to most figure panels: a) foci appear in all stages of the representative images in 4D, it is thus not entirely clear that HSP42 phosphorylation state is associated with complete solubilization. Quantification of number of cells with foci, focus size, the number of foci per cell, etc. is necessary here. b) Similarly, quantification of the images in 4D, 5E, and 5F showing the number of cells with with this phenotype is essential to reach a conclusion."

As the reviewer pointed out, the missing error bars for Pindex trajectories and phosphorylation abundance were added to Figure 3, 4, S3, S4 and S5. Moreover, to address the question concerning the microscopic images errors, we chose to quantify the heterogeneity score (covariant coefficients) for the fluorescence microscopic images. This score is a reflection of how much fluorescent signal is localized or diffused at the cellular level. Distribution of heterogeneity score in a population of cells reveals the diversity in behavior of a protein. We explained the method as follow:

Fluorescence heterogeneity score

To measure cellular heterogeneity score, fluorescent images were opened in ImageJ and cell peripheries were drawn by hand to analyze fluorescence signals of single cells. Heterogeneity scores are the fluorescence signal covariant coefficients, obtained by dividing the standard variation by the mean signal.

Shown in Figure 3D, 4D, 5D, 5G, S6A, is the distribution of single cell measurements of heterogeneity. With higher score meaning more focused signal, and low score meaning more diffuse signal, we can easily appreciate the distribution of the localization among a population.

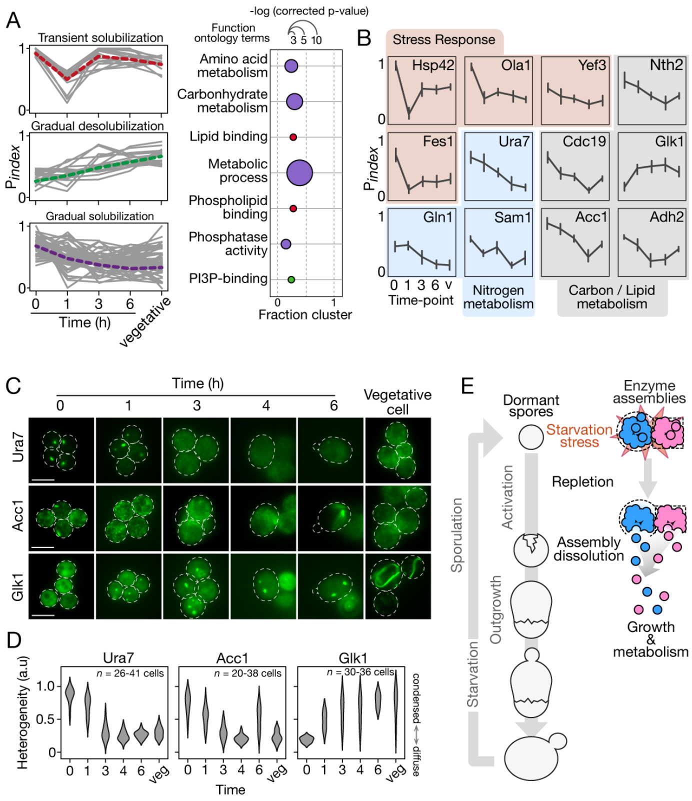

Figure 3 - Solubility changes reflect metabolism activation and mimic stress relief during germination.

A) Enrichment for gene ontology terms in each dynamically changing solubility cluster. Red, transiently solubilizing cluster; Green, gradual desolubilization cluster; Purple, gradual solubilization cluster. The position on the x-axis indicates the portion of the proteins in a cluster

assigned to a GO term, and size of the bubble is scaled to the -log (*p*-values) from a hypergeometric test.

B) Individual P_{index} trajectory for representative proteins through germination. Bar represents standard variation. Proteins are clustered by function; Red, stress response proteins; Blue, Nitrogen metabolism proteins; Gray, Lipid and carbon metabolism proteins. Error bars represent standard deviation of three replicates.

C) Representative fluorescence microscopic images of spores expressing the indicated proteins tagged with GFP during germination. Top to bottom, Acetyl-CoA carboxylase Acc1 (Lipid biosynthesis), CTP synthase Ura7 (pyrimidines synthesis) and Glucokinase Glk1 (glycolysis). The Glk1 foci formation and the dissolution of Acc1 and Ura7 foci in course of germination support that dormancy in spores is analogous to a stress state and germination alleviates this state. Dotted lines indicate cell contour determined by brightfield images. Scale bars represent 5μm.

D) Measure of cellular heterogeneity (coefficient of variation) of the fluorescent proteins in spore at the indicated time after exposure to rich medium or in vegetative cells. Between 20 and 41 cells were analyzed at each time-points.

E) Schematics highlighting effects on protein solubility of nutrient starvation and repletion during sporulation and germination, respectively. Pink and blue assemblies represent assemblies of enzymes needed for growth and metabolism during dormancy, which disassemble (pink and blue circles) during germination.

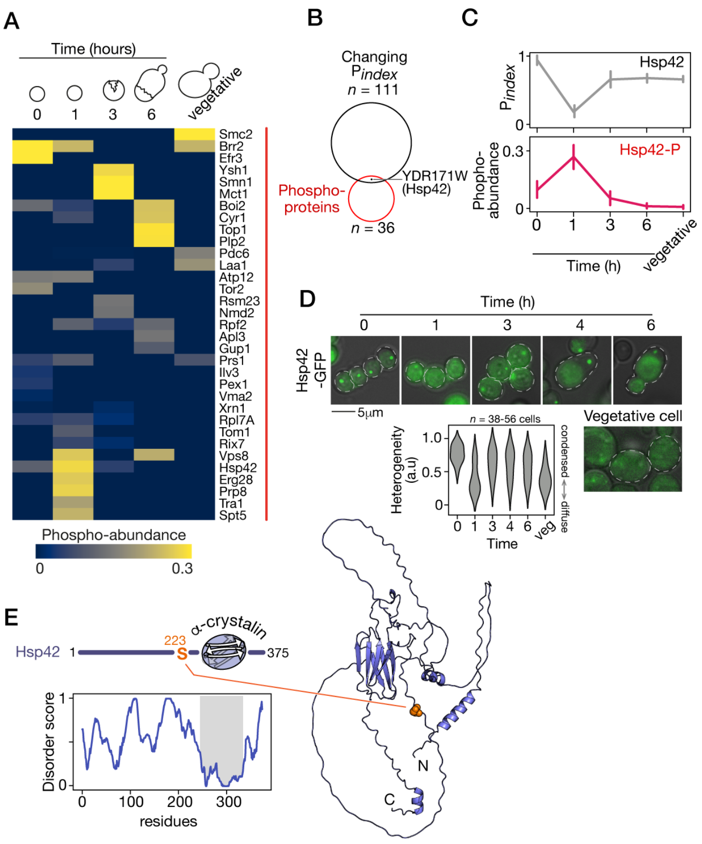

Figure 4 - Hsp42 phosphorylation at S223 is synchronized with its transient solubilization.

A) Relative abundance of the 36 phosphoproteins to the total abundance of each protein through germination.

B) Hsp42 is phosphorylated during germination and changes solubility. See Figure S3 for additional information.

C) Hsp42 is the only protein with dynamic solubility profile during germination that correlates with its dynamic phosphorylation, here at S223. Error bars represent standard deviation of three replicates.

D) Representative fluorescence microscopic images of spores expressing Hsp42-GFP at the indicated time after the induction of germination. Dotted lines represent cell contour. Scale bar represents 5μm. Plot shows the cellular Hsp42-GFP heterogeneity score in spore at the indicated time after exposure to rich medium or in vegetative cells.

E) Left, Disorder profile of Hsp42, predicted by Metapredict, shows the predicted structured ACD domain, and flanking disordered N- and C-terminal region. Right, predictedHsp42 structure. The S223 highlighted in orange is located in a disordered region.

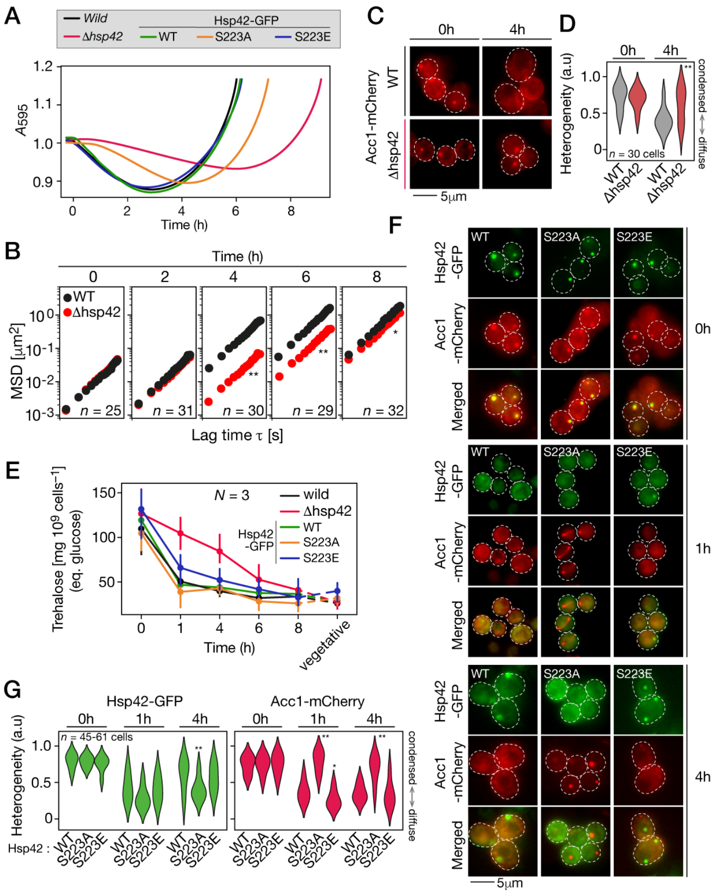

Figure 5 - Active and phosphorylated Hsp42 is required for normal germination dynamics.

A) Optical density of pure spore cultures of the indicated strains as a function of time following exposure to germination conditions. Shown are the mean values of three replicates. OD drop of the Δhsp42 spores is strongly delayed, indicating that germination is inhibited or slowed down. Spores expressing S223A Hsp42 mutant show a slight delay in germination, while germination of spores expressing S223E Hsp42 mutant is indistinguishable from that of wild spores.
B) Ensemble mean square displacement of μNS-GFP in WT and Δhsp42 spores at the indicated time after exposure to rich medium. At each time point for each strain, 25 to 35 particles, corresponding to the same number of cells, were tracked. Kruskal-Wallis test, ** indicates p-value < 0.0001, * indicates p-value < 0.01.
C) Fluorescence microscopy images of wild type (top) or ΔHsp42 spores expressing Acc1-mCherry at the indicated time after exposure to germination conditions. Scale bar represents 5μm.
D) Cellular Acc1-mCherry heterogeneity score in WT or Δhsp42 spores at the indicated time after exposure to rich medium.
E) Trehalose content (measured in equivalent of glucose concentration) in spores at the indicated time after exposure to rich medium, and in vegetative yeasts. Error bars represent standard deviation of three replicates.
F) Fluorescence microscopy images of spores expressing either WT, S223A or S223E mutant Hsp42-GFP and Acc1-mCherry at the indicated time after exposure to germination conditions. Dotted lines represent cell contour. Scale bar represents 5 μm. Expression of S223A Hsp42-GFP mutant shows delay in Acc1 foci dissolution in spores.

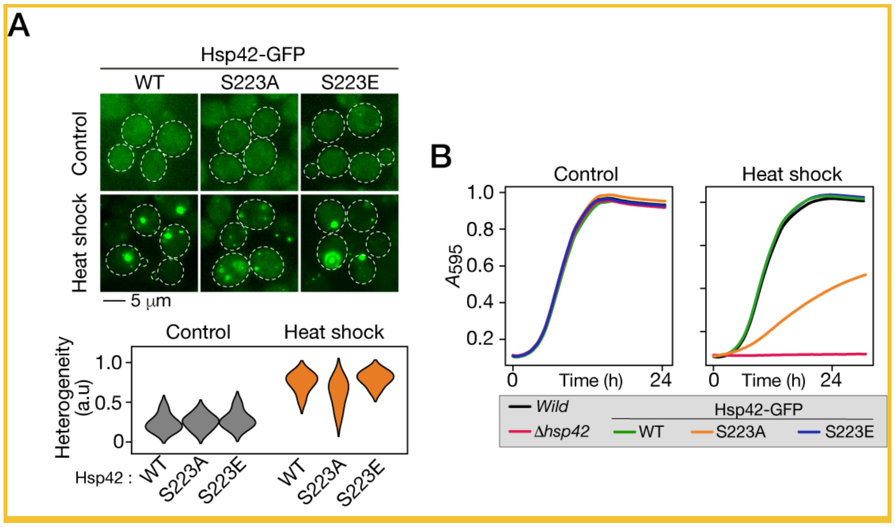

Figure S6 - Stress activation of Hsp42 through its phosphorylation on S223, related to Figure 5.

A) Fluorescence microscopic images of WT or mutant Hsp42-GFP expressing cells, in control conditions (top) or after a heat shock (bottom). Dotted lines represent cell contour. Scale bar represents 5 μm. Bottom, cellular Hsp42-GFP heterogeneity measure. The S223A Hsp42 mutant shows smaller and fainter aggregates in cells, and lower heterogeneity score compared to WT and phosphomimetic mutant. Heat shock at 50˚C for 10 minutes.
B) Growth curves of vegetative cells of the indicated strains after a heat shock (right) or a mock treatment at control temperature (left). Shown are the mean values of three replicates. The

S223A Hsp42 mutant shows intermediate heat-shock resistance between the WT (and S223E mutant) and *HSP42* deleted cells. This confirms that the phosphorylation of Hsp42 at this site is important for its function.

“6. Linking Hsp42 phosphorylation and activity to phenotype. From the data presented the correlation between the Hsp42 phosphorylation state and solubility remains a bit unclear to me. First, the authors showed an inverse correlation between the P index of HSP42 (measured by the MS peptides) and the Phosphopeptide detected (without Phospho-enrichment in the experiment) i.e., when the protein is more soluble the phosphorylation on S223 is increased. This inverse correlation suggests an importance of the phosphorylation on S223 for the solubility of HSP42. However, it is the phospho-dead mutant (S223A) rather than the phosphomimetic mutant (S223E) that is solubilized after 4h of germination in Fig. 5F. At the same time, at time 0h Hsp42(SS223E) forms assemblies, suggesting that phosphorylation may not impede their formation either. The authors need to clarify this. The quantification I suggest may help, but considering some alternative mechanistic models may also be useful, as would a similar tighter time course for this experiment that corresponds to the points chosen for the MS data (and presented in Fig. 4D). As presented, currently presented it is unclear why S223E isn't the wild-type allele. It seems better at all activities examined.”

We agree that inverse correlation of Hsp42 phosphorylation and Hsp42 solubilization suggest that phosphorylation might be important for solubilization. However, we reported that the S223A mutant solubilizes during germination, and S223E mutant forms condensate in spores, suggesting that phosphorylation has contradictory effects on solubility. In a way to clarify the function of S223 phosphorylation, we showed microscopic images of spores expressing mutant Hsp42 at one hour time-point. This result reveals that solubilization of Hsp42 happend regarless of the mutation tested. This result corroborates the absence of mutational effect of Hsp42 on the mobilization of trehalose (Figure 5E). Together, we believe these results indicated Hsp42 has a function independent of its phosphorylation on S223 that is in relation with its solubilization and trehalose mobilization. We added these results in Figure 5.

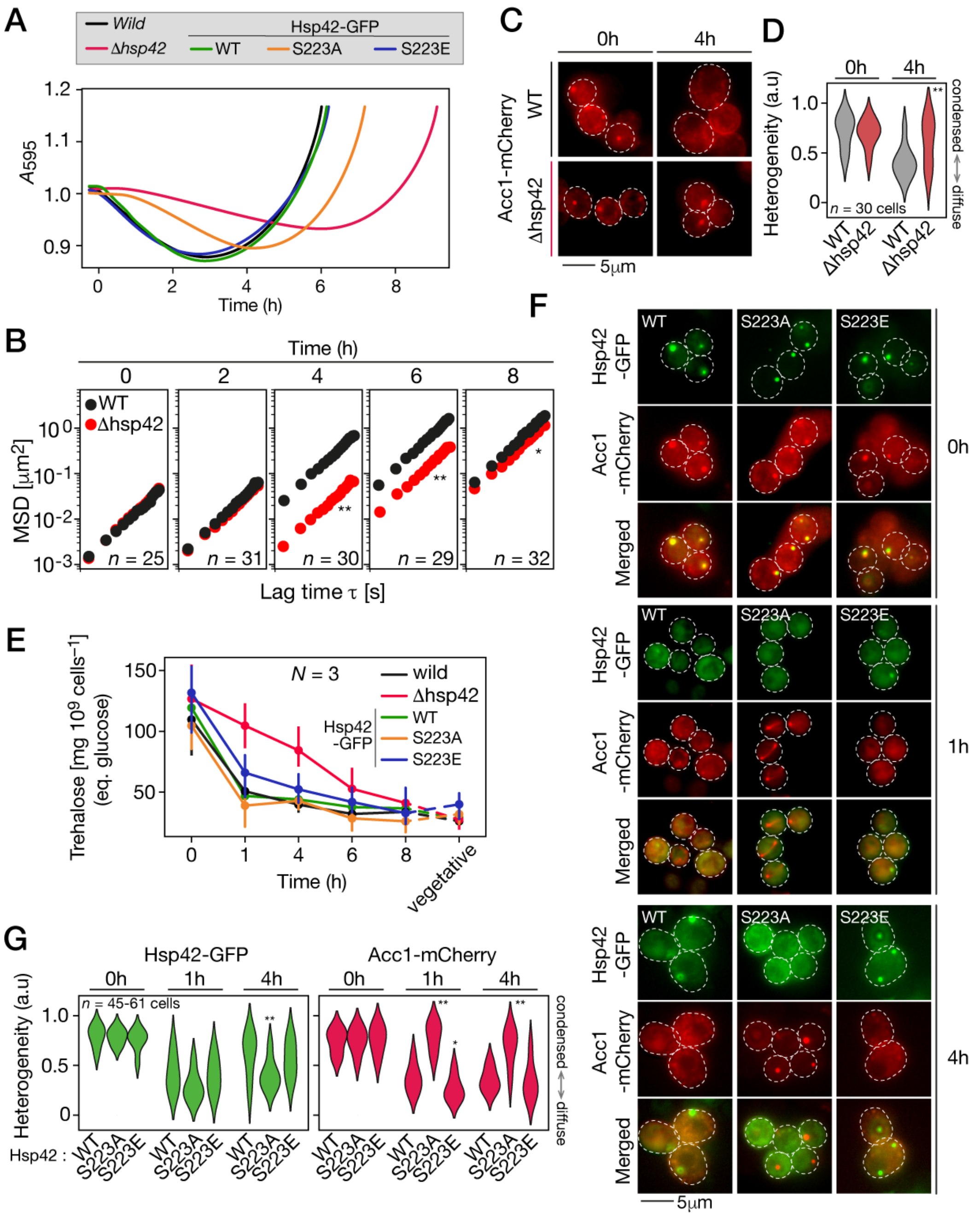

Figure 5 - Active and phosphorylated Hsp42 is required for normal germination dynamics.

A) Optical density of pure spore cultures of the indicated strains as a function of time following exposure to germination conditions. Shown are the mean values of three replicates. OD drop of the Δhsp42 spores is strongly delayed, indicating that germination is inhibited or slowed down. Spores expressing S223A Hsp42 mutant show a slight delay in germination, while germination of spores expressing S223E Hsp42 mutant is indistinguishable from that of wild spores.

B) Ensemble mean square displacement of μNS-GFP in WT and Δhsp42 spores at the indicated time after exposure to rich medium. At each time point for each strain, 25 to 35 particles,

corresponding to the same number of cells, were tracked. Kruskal-Wallis test, ** indicates p-value < 0.0001, * indicates p-value < 0.01.
C) Fluorescence microscopy images of wild type (top) or ΔHsp42 spores expressing Acc1-mCherry at the indicated time after exposure to germination conditions. Scale bar represents 5μm.
D) Cellular Acc1-mCherry heterogeneity score in WT or Δhsp42 spores at the indicated time after exposure to rich medium.
E) Trehalose content (measured in equivalent of glucose concentration) in spores at the indicated time after exposure to rich medium, and in vegetative yeasts. Error bars represent standard deviation of three replicates.
F) Fluorescence microscopy images of spores expressing either WT, S223A or S223E mutant Hsp42-GFP and Acc1-mCherry at the indicated time after exposure to germination conditions. Dotted lines represent cell contour. Scale bar represents 5 μm. Expression of S223A Hsp42-GFP mutant shows delay in Acc1 foci dissolution in spores.

Following the sentence “in these cells, Acc1 foci failed to disassemble (Figure 5F, 5G)” we added the underlined text to discuss the solubilization of Hsp42 and the role of its phosphorylation.

Altogether, activation of Hsp42 through phosphorylation on S223 appears to be crucial to disassembly of low solubility protein and germination progression. Moreover, our results revealed a surprising contribution of phosphorylation to behavior of Hsp42 in germination. Both non-phosphorylatable S223A or phosphomimetic S223E Hsp42 mutants were solubilized at one-hour time-point, then at a later time-point (four hours), S223A mutant remained diffuse in the cytoplasm while WT and S223E mutant formed foci (Figure 5F). These results refine our hypothesis about the role of this phosphorylation on Hsp42 solubilization: they suggest that some processes independent of Hsp42 phosphorylation on S223 are involved. Among these processes is the quick mobilization of trehalose. Our results revealed the presence of Hsp42 is crucial for trehalose mobilization, but contrastingly, it occurs regardless of the Hsp42 mutant tested (Figure 5E). Altogether, our results highlight many functions of the molecular chaperone Hsp42 for germination progression, some of which involve its phosphorylation on S223.

“Additionally, the authors cite Kanshin et al. (2015): "The abundance of this phosphorylation was found to increase in cells following exposure to heat" when showing that heat shocked HSP42 is found in assemblies (Fig 5B). Again, these data suggest that phosphorylation may occur even while HSP42 is in assemblies, although solubility and focus formation may not be correlated given the diffraction limit in these experiments. The authors may want to test whether Hsp42 also becomes insoluble during heat shock using their centrifugation protocol.”

Following the sentence “Hsp42-dependent spatial protein organization is crucial for cellular fitness, and lack in foci formation results in a significant delay when recovering from stationary phase (Liu et al., 2012)” we added the mention of a previous work using sedimentation that reports low solubility of Hsp42 during stress.

Notably, sedimentation of Hsp42 was found to increase overtime in yeast faced with heat stress suggesting the trigger of its chaperon function, through a physical separation technique similar to ours (Wallace et al. 2015)

"7. I would suggest adding some annotation in Fig 1D to guide the reader"

We agree that annotations on the TEM panel are missing but would be beneficial to the reader. As suggested, we added annotation in Figure 1 TEM panel.

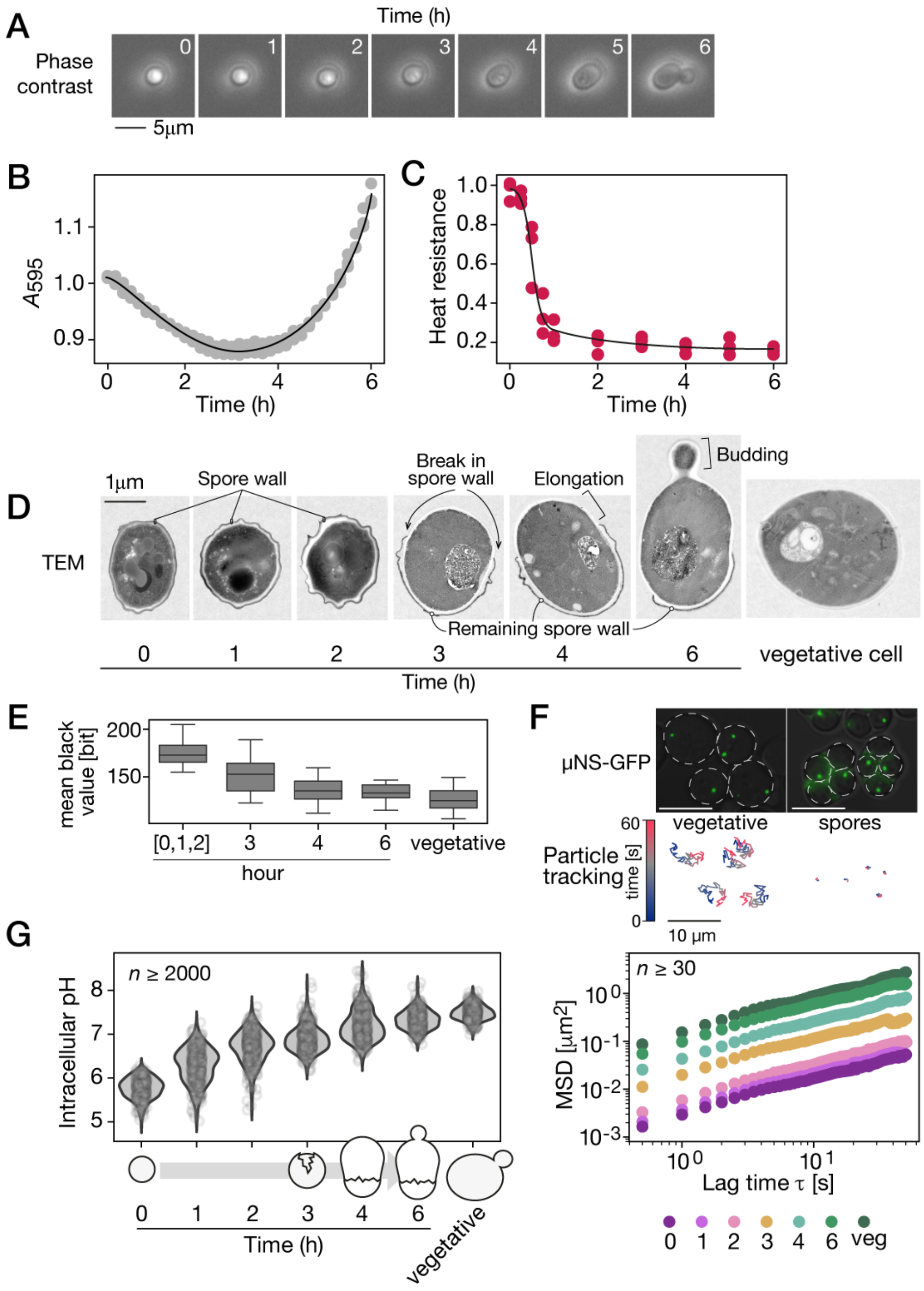

Figure 1 - The cytoplasm of dormant spores displays high rigidity and density and is an acidic environment.
A) Phase-contrast microscopic images of ascospore (same cell followed through time) at the indicated time after exposure to rich media, which activates germination. Scale bar represents 5 µm.
B) Optical density (A_{595}) and C) heat resistance of pure spore cultures through time after exposure to rich media. Heat resistance is the ratio of growth after a heat shock at 55˚C for 10 minutes to growth without heat treatment. Experiments were performed in triplicate and values for individual replicates are shown.
D) Representative transmission electron microscopic (TEM) images of spores at the indicated time after exposure to rich medium, and of a vegetatively growing yeast cell (vegetative). Cells were prepared and stained at the same time. Imaging was performed on a single layer. The scale bar represents 1 µm. See Figure S1A for more examples.
E) Mean black level of spore cytosol at the indicated time after exposure to rich medium and of vegetative yeast. Spores at 0, 1 and 2-hour time-points are merged into a single category since they are indistinguishable from one another.
F) Top, microscopic images of µNS-GFP particles in vegetative yeasts and spores. Underneath is the corresponding 1 minute trajectories of the particles. Color indicates time scale. Scale bar represents 10 µm. Bottom, ensemble Mean Squared Displacement (MSD) of µNS-GFP particles in spores at the indicated time after exposure to rich medium and in vegetative cells.
G) Intracellular pH measured at the indicated time point after germination induction, and in exponentially growing cells. Measurements in at least 2000 cells are shown at each time point.

“9. The authors refer to the scheme of the LC-MS/MS in page 7 as Figure 1A instead of Figure 2A”

We corrected this mistake.

8. It may be worth more explicitly making the point that the changes in solubility are mostly *not* about phosphorylation given the data in Fig. 4B

We explicitly made this point by adding the sentence in the result section:
“The small intersection between these clusters presuppose that phosphorylation is not a primary factor driving the change in protein solubility.”

Reviewer 3

“1) There is an overall difficulty with communication of the data because of the vocabulary used in the "yeast" field. The current presentation is confusing and needs to have more specificity/clarity if the work is going to appeal to a broad readership. It begins with the title:

"Exit of spore dormancy transforms the yeast cytoplasm and the solubility of its proteome"

Does "yeast" in this case mean S. cerevisiae? Or does it mean the cell type "yeast?" If it means the budding yeast S. cerevisiae, then that needs to be indicated or somehow clarified. If it means the cell type known as yeast, then the title is unclear because exiting spore dormancy transforms the spore cytoplasm to facilitate the transition into a yeast.

Removing "yeast cytoplasm" from the title would be helpful. Suggestions to consider:

Exit of dormancy by fungal spores transforms the cytoplasm and the solubility of the proteome

Exit of dormancy by spores of S. cerevisiae transforms the cytoplasm and the solubility of its proteome

Breaking spore dormancy in S. cerevisiae transforms the cytoplasm and the solubility of its proteome

Breaking dormancy in spores of budding yeast transforms the cytoplasm and the solubility of its proteome"

As suggested here, we change the previous title "Exit of spore dormancy transforms the yeast cytoplasm and the solubility of its proteome" to
Breaking dormancy in spores of budding yeast transforms its cytoplasm and the solubility of its proteome

"2) Along these same lines, the manuscript is very confusing with respect to the natures and states of the cell types in question. It is sometimes difficult to discern whether the population being discussed is dormant spores, germinating spores, germinated spores, vegetatively growing yeast, or quiescent yeast. It might seem like semantics, but in this case, it is critical to have clarity on cell type and growth state so that the data can be interpreted correctly.
a. Part of the challenge may stem from habits in the Saccharomyces field, which tends to refer to "spores and cells" or "spores and yeast cells," and this is extremely confounding because spores are cells. A workable solution is to simply refer to the two cell types in question (spores and yeast) and avoid the use of the word "cell" altogether whenever possible. One can then use a descriptor to modify the type of spore or yeast being discussed. For example, "germinating spore" or "replicating yeast." Because there are relatively quiescent states for yeast (such as stationary phase), the state of the yeast should be made

clear to readers (because not all yeast are growing vegetatively). It is clear that the authors have attempted to differentiate among the different cell types and states in many instances, but this should be revisited in the text and fully addressed with consistent labels and descriptors. It doesn't really help to use the specific term "spores" and the
general term "vegetative cells" when making comparisons. "Vegetative cells" has different meanings across systems from bacteria to multicellular organisms, so it would be helpful to use more specificity in this manuscript. Replace "vegetative cells" with "yeast" or "vegetative yeast" or "growing yeast" as needed."

As suggested, to avoid confusion in respect to cell type nomenclature, we changed the term "vegetative cells" across the manuscript to "vegetative yeasts".

"3) Comments on Abstract: is "Yeasts" is unclear. Not all yeasts produce spores. "Many yeasts produce . . ." followed by "We show that spores of S. cerevisiae exhibit . . ." would be more clear."

As suggested, we clarified the abstract by adding the mention "Many yeast produce dormant spores…" and specified in the next sentence "We show that spores of *Saccharomyces cerevisiæ* exhibit…"

"4) Comments on Introduction
a. paragraph at the top of page 4 needs to make it clear that "yeast" in this case is S. cerevisiae. ". . . cell cytoplasm also come from yeast, such as S. cerevisiae, responding to acute stresses. . ."

As suggested, we clarified the sentence in the introduction by adding the underlined text: "…responsiveness of the biophysics of the cell cytoplasm also come from yeast, such as *S. cerevisiæ*, responding to acute stresses".

"b. last paragraph needs to make it clear what system and spore type is being used. "Here, we therefore examine the biophysical properties of budding yeast ascospores and the changes that occur during the breaking of dormancy to unveil the molecular processes that support this critical life-history cellular transition."

As suggested, we clearly mentioned the yeast and spore systeme used in this work by changing the underlined sentence in the last paragraph of introduction:
"Here, we therefore examine the biophysical properties of dormant budding yeast ascospores and the changes that occur during dormancy breaking to unveil the molecular processes which support this critical life-history cell transition."

"5) Comments on Figure 1 data and results
a. Legend title - change "cells" to "spores"

b. 1A: indicate that the germinating spore is the same one over the time course (not representative images)"

As suggested, we changed the figure 1 title from "The cytoplasm of dormant cells displays high viscosity and is an acidic environment." to "The cytoplasm of dormant spores displays high rigidity and density and is an acidic environment."
We also change Figure 1A legend to state "Phase-contrast microscopic images of ascospore (same cell followed through time) at the indicated time…"

"c. 1B: p. 5 in reference to Figure 1B. It would be helpful comment on/speculate about why the OD595 increases after 3 hours of germination. Does refractility increase? Or is this due to an increase in optical density due to budding?"

We reckon that a complete description of the OD curves is needed. To clarify the OD curves, we added the following sentence in the first paragraph of results: "... with the minimal values reached about 3 hours after induction (Figure 1B). Then subsequent growth leads to an increase of optical density."

"d. 1D: Apparent density in TEM can be heavily influenced by the process of fixation. Were all of these images selected from samples prepared, fixed, and imaged at the same time? Please indicate - particularly if a comparison is going to be made across images."

To clarify the way cells were prepared prior to imaging, we added the underlined statement in Figure 1 legend:
D) Representative transmission electron microscopic (TEM) images of spores at the indicated time after exposure to rich medium, and of a vegetatively growing yeast cell (vegetative). Cells were prepared and stained at the same time. Imaging was performed on a single layer. The scale bar represents 1 μm. See Figure S1A for more examples.

"e. 1D: While the text indicates that "the spore cytoplasm appears darker in TEM in comparison to a vegetative cell, which suggests a denser cytosol. . .," this appears to be the case in the images in 1D but not the case in S1A. A darker spore cytoplasm is not supported by the images shown.
f. Is there a method to quantitate the apparent density per area? If spores getting bigger over time, one might expect the apparent density to decrease simply due to the same electron density being spread over a larger area. Is there anything in these images to counter that argument?"

We agreed with the reviewer that the great variation in cytoplasmic density appears conflicting with our results. To answer this issue, we quantified the pixel density in microscopic images, and presented the mean pixel count as a function of time in germination in Figure 1E.

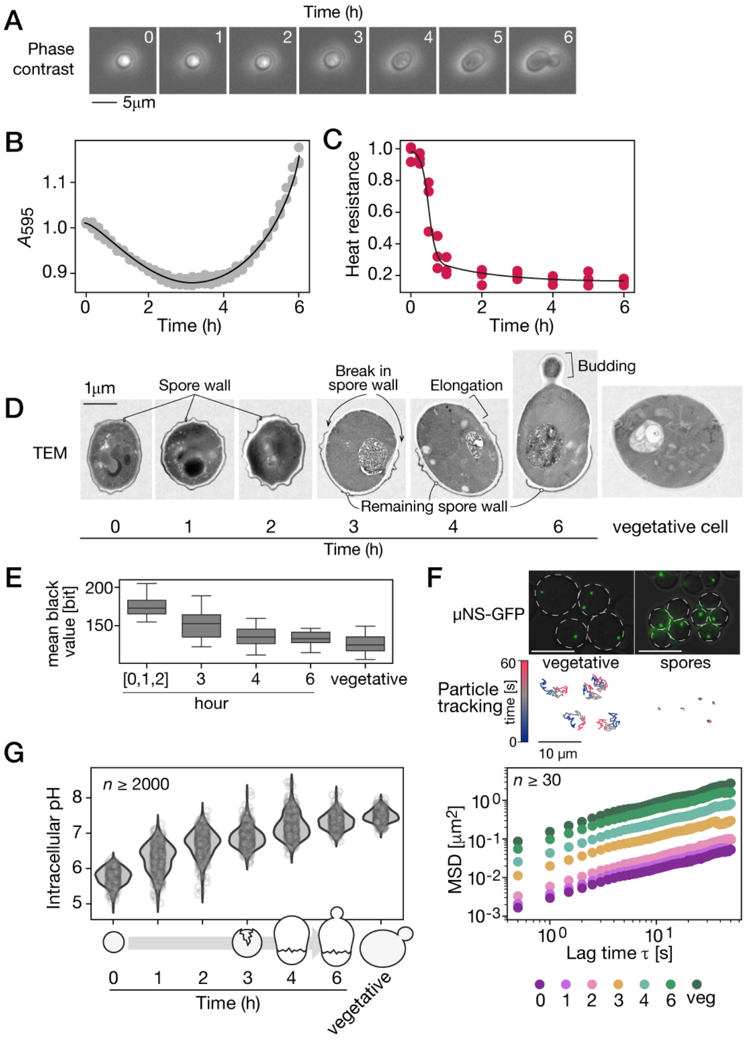

Figure 1 - The cytoplasm of dormant spores displays high rigidity and density and is an acidic environment.

A) Phase-contrast microscopic images of ascospore (same cell followed through time) at the indicated time after exposure to rich media, which activates germination. Scale bar represents 5 µm.

B) Optical density (A_{595}) and C) heat resistance of pure spore cultures through time after exposure to rich media. Heat resistance is the ratio of growth after a heat shock at 55˚C for 10 minutes to growth without heat treatment. Experiments were performed in triplicate and values for individual replicates are shown.

D) Representative transmission electron microscopic (TEM) images of spores at the indicated time after exposure to rich medium, and of a vegetatively growing yeast cell (vegetative). Cells were prepared and stained at the same time. Imaging was performed on a single layer. The scale bar represents 1 µm. See Figure S1A for more examples.

E) Mean black level of spore cytosol at the indicated time after exposure to rich medium and of vegetative yeast. Spores at 0, 1 and 2-hour time-points are merged into a single category since they are indistinguishable from one another.

F) Top, microscopic images of μNS-GFP particles in vegetative yeasts and spores. Underneath is the corresponding 1 minute trajectories of the particles. Color indicates time scale. Scale bar represents 10 μm. Bottom, ensemble Mean Squared Displacement (MSD) of μNS-GFP particles in spores at the indicated time after exposure to rich medium and in vegetative cells.
G) Intracellular pH measured at the indicated time point after germination induction, and in exponentially growing cells. Measurements in at least 2000 cells are shown at each time point.

g. The text indicates that "Spores have a different cell organization." A clearer description would be to refer to "cytoplasmic organization."

To clarify the description of our results we change the text as suggested:
"Spores have a different cytoplasmic organization. This is shown by the membranous…"

"h. The authors also need to take care to refer to visible features changing as correlating with other properties - not as a consequence or cause of. For example, "Visible cytoplasmic organization changed after about three hours of germination, which correlated with a drop in heat resistance comparable to levels seen in vegetatively growing yeast."

As suggested, we refer to correlation in this results section:
"…indistinguishable from dormant cells. Visible cytoplasmic organization changed after about three hours of germination, which correlated with a drop in heat resistance comparable to levels seen in vegetatively growing yeast (Figure 1D). At this time point…"

"i. It is not clear what the authors are intending to communicate with the sentence, "These observations suggest that the spore cytosol organization is timely modulated in the course of germination and return to vegetative growth." Perhaps -- Cytosolic reorganization temporally coincides with a return to vegetative growth?"

We clarified our statement as suggested:
" These observations suggest that transformation of the physical nature of the cytosol environment coincides with germination and return to vegetative growth ".

j. It is difficult to argue that the viscosity experiments in 1E and 1F are "validating" the observation that cytoplasmic density is decreasing, when the evidence for decreasing density during germination is an observation based on TEM. It would be better to present the TEM data as suggestive, form a hypothesis, and present the viscosity and pH experiments as tests of the hypothesis.

We changed our presentation from "To validate our observations that the density of the cytoplasm decreases during germination, we quantified…" to "To test the cytosol physical

properties during germination, we quantified its dynamic through the examination of macromolecular motion. We expressed the reovirus…"

k. It is important that the authors clarify what was known about the behavior of μNS-GFP prior to these studies, what they showed for their specific strains, and what is newly shown in this manuscript.

As suggested, we added sentences to explain previous results using μNS particle and compare with our results:
"Other experiments using μNS as tracer particles reported mean squared displacement (MSD) at 1 second lag-time in the order of 10^{-1} μm^2 (Munder et al. 2016), which is in the range of our results. While energy deprivation in yeast reduces MSD of tracer particles by less than one order of magnitude (Munder et al. 2016; Joyner et al. 2016), we reported that particles have motion two orders of magnitude lower than in vegetative yeast. These results highlight that dormant spores have an exceptionally dense and rigid cytosol".

"l. The reference to Figure S2 on the bottom of page 5 appears to be incorrect. Where are the μNS-GFP single molecule tracking data?? Relevant data appear to be in Figure S1B and S1C, but there isn't enough information to suss out the progression of data collection and corrections for size effect."

We agree that single particle data are important but missing from our results. As suggested, we added single particle tracking data.
In Figure 1F (Top), we show μNS-GFP microscopic images and 1 minute tracking of these particles.

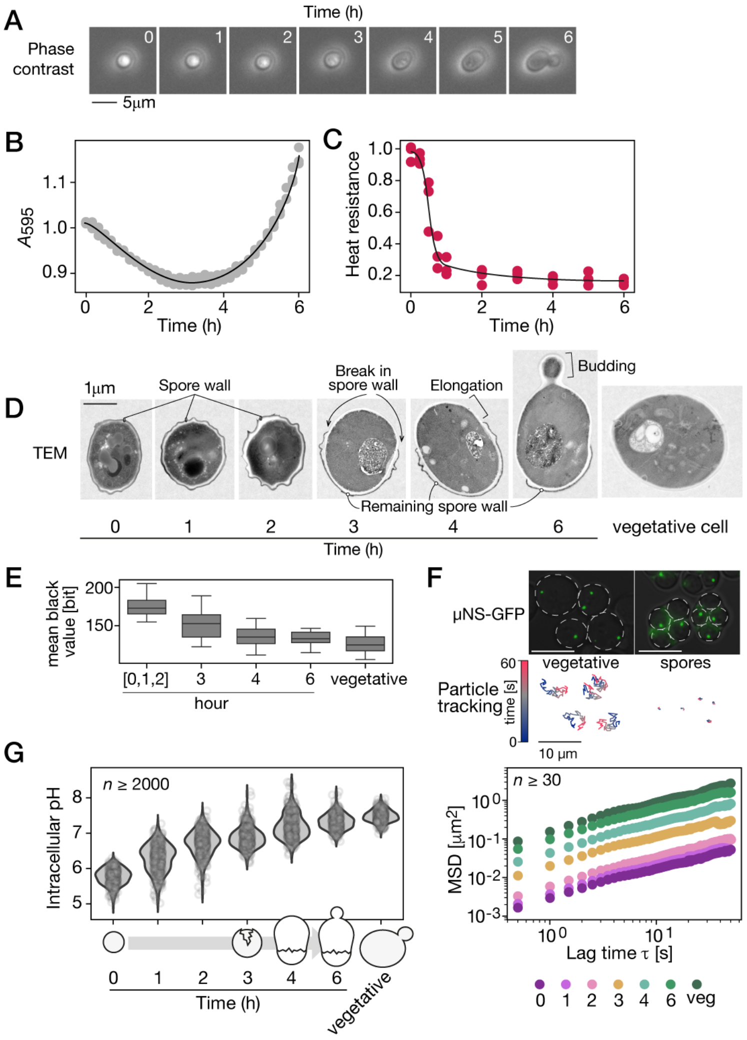

Figure 1 - The cytoplasm of dormant spores displays high rigidity and density and is an acidic environment.

A) Phase-contrast microscopic images of ascospore (same cell followed through time) at the indicated time after exposure to rich media, which activates germination. Scale bar represents 5 µm.

B) Optical density (A_{595}) and C) heat resistance of pure spore cultures through time after exposure to rich media. Heat resistance is the ratio of growth after a heat shock at 55˚C for 10 minutes to growth without heat treatment. Experiments were performed in triplicate and values for individual replicates are shown.

D) Representative transmission electron microscopic (TEM) images of spores at the indicated time after exposure to rich medium, and of a vegetatively growing yeast cell (vegetative). Cells were prepared and stained at the same time. Imaging was performed on a single layer. The scale bar represents 1 µm. See Figure S1A for more examples.

E) Mean black level of spore cytosol at the indicated time after exposure to rich medium and of vegetative yeast. Spores at 0, 1 and 2-hour time-points are merged into a single category since they are indistinguishable from one another.

F) Top, microscopic images of μNS-GFP particles in vegetative yeasts and spores. Underneath is the corresponding 1 minute trajectories of the particles. Color indicates time scale. Scale bar represents 10 μm. Bottom, ensemble Mean Squared Displacement (MSD) of μNS-GFP particles in spores at the indicated time after exposure to rich medium and in vegetative cells.
G) Intracellular pH measured at the indicated time point after germination induction, and in exponentially growing cells. Measurements in at least 2000 cells are shown at each time point.

In Figure S1C we show single particle Mean square displacement data at each time-point in germination.

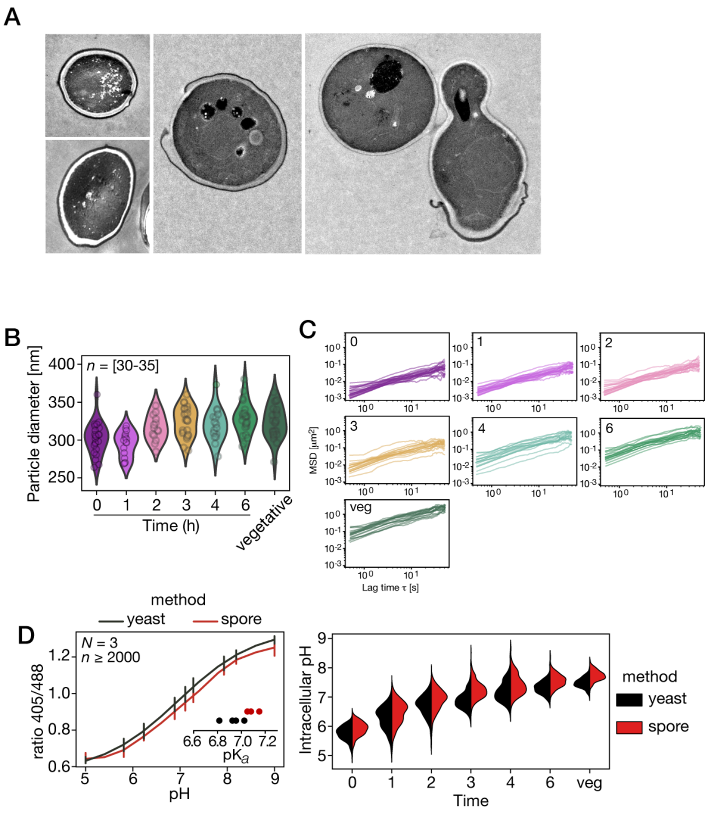

Figure S1 - μNS-GFP particle size and Mean square displacement (MSD) during germination; calibration curve of pHluorin emission ratio, related to figure 1.
A) Transmission electron microscopy images of spores at the indicated time after exposure to rich medium. Scale bar represents 1 μm.
B) Size of individual particles tracked at each time point during germination and in vegetatively growing cells. At least 30 particles were tracked at each time point.
C) Mean square displacement of individual particles tracked at the indicated time after germination induction.
D) Left, intracellular pH calibration curves determined using vegetative yeast (black) or spores (red). Although the curve using spores is slightly more basic, measurement of pH in germination (right) using either curve shows that spores are acidic and that intracellular pH increases steadily during germination. Logistic function fitted to the data from vegetative cells was used for Figure 1G. Error bars represent standard deviation of three replicates.

“m.   It is unclear what particle size difference the authors are referring to at the top of page 6 (and apparently visible in Figure S1A). Are there μNS particles shown somewhere? No information in the legend for S1A.”

The reviwer made a good point that our data do not show a clear size difference in particle size (therefore that statement was removed), and because our tracking experiments do not cover diverse particle size, use of viscosity is not adequate. We changed our results to refer to “particle mobility” and “fluidity” of the cytoplasm.
Example of these changes:
“The tracking of single particles revealed their lower mobility in dormant spores compared to vegetative yeast (Figure 1F). These measurements suggest that dormant spore cytoplasm is highly rigid or dense. Particle motion remained low during the first two hours of germination, then increased gradually from hatching (3 hour time-point) until the end of germination (Figure 1F). At bud emergence, the motion of μNS particles is close to that measured in vegetative yeast.”
We changed Figure 1F and 5B to show Mean Squared Displacement instead of viscosity There is no size correction to be done anymore.

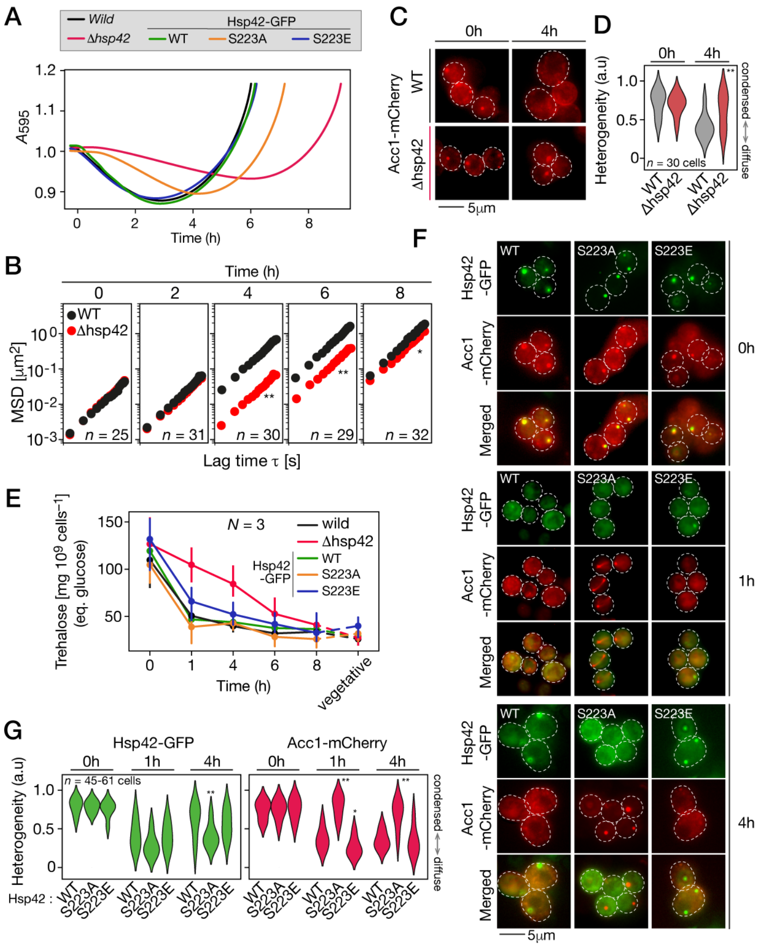

Figure 5 - Active and phosphorylated Hsp42 is required for normal germination dynamics.

A) Optical density of pure spore cultures of the indicated strains as a function of time following exposure to germination conditions. Shown are the mean values of three replicates. OD drop of the Δhsp42 spores is strongly delayed, indicating that germination is inhibited or slowed down. Spores expressing S223A Hsp42 mutant show a slight delay in germination, while germination of spores expressing S223E Hsp42 mutant is indistinguishable from that of wild spores.
B) Ensemble mean square displacement of μNS-GFP in WT and Δhsp42 spores at the indicated time after exposure to rich medium. At each time point for each strain, 25 to 35 particles, corresponding to the same number of cells, were tracked. Kruskal-Wallis test, ** indicates p-value < 0.0001, * indicates p-value < 0.01.
C) Fluorescence microscopy images of wild type (top) or ΔHsp42 spores expressing Acc1-mCherry at the indicated time after exposure to germination conditions. Scale bar represents 5μm.
D) Cellular Acc1-mCherry heterogeneity score in WT or Δhsp42 spores at the indicated time after exposure to rich medium.

E) Trehalose content (measured in equivalent of glucose concentration) in spores at the indicated time after exposure to rich medium, and in vegetative yeasts. Error bars represent standard deviation of three replicates.
F) Fluorescence microscopy images of spores expressing either WT, S223A or S223E mutant Hsp42-GFP and Acc1-mCherry at the indicated time after exposure to germination conditions. Dotted lines represent cell contour. Scale bar represents 5 µm. Expression of S223A Hsp42-GFP mutant shows delay in Acc1 foci dissolution in spores.

“n. The µNS-GFP measurements shown do not confirm "what we see in the TEM images." They are simply consistent with high cytoplasmic density in dormant spores and lower cytoplasmic in germinating spores with the lowest cytoplasmic density occurring in growing yeast.”

This paragraph was reformulated, and the sentence “These measurements confirmed what was seen in the TEM images: dormant spore cytoplasm is the densest among all of these stages” was replaced with “These measurements suggest that dormant spore cytoplasm is highly rigid or dense. Particle motion..”

“o. p. 6 first paragraph last sentence should read, "These observations are in the same range of viscosity as ascospores of the dimorphic fungus Talaromyces macrosporus, which are characterized by unusually high cytoplasmic viscosity.”

This sentence was changed to “These observations are in agreement with previous work on the fungi *Talaromyces macrosporus*, where spores were found to be characterized by high viscosity (Dijksterhuis et al., 2007)”
“p. p. 6 last paragraph - The intent of the sentence, "These conditions are timely modulated during the germination and return to vegetative growth" is not at all clear. The phrase "timely modulated" has no meaning.”

As suggested, we clarified this sentence
“…a highly rigid and acidic cytoplasm. These conditions are modulated during the germination and return to vegetative growth. These intracellular properties…”

“6) Comments on Figure 2 data and results
a. Reference to Figure 1A at the top of page 7 is incorrect. Should be Figure 2A.”

This mistake was corrected.

“b. p. 7 first paragraph "Values for these 895 proteins range from 0 to 1. Zero indicates that the protein was detected only in the supernatant, and 1 indicates that the protein was detected only in the pellet."

As suggested, we changed the sentence

“Values for these 895 proteins range from 0 to 1. Zero indicates that the protein was detected only in the supernatant, and 1 indicates that the protein was detected only in the pellet.”

“c. 2B: The authors indicate that LC-MS/MS was carried out on fractions from "5 time points during germination." This is incorrect according to their data. There were four times points during germination (0, 1, 3, and 6 hours) and one sample of vegetatively growing yeast. This needs clarified in the text and figure legend (unless the last timepoint is a culture of germinated spores that were left to grow as a yeast culture. In that case, it should be referred to as its timepoint of harvest after initiation of germination).”

We clarified this sentence by saying instead:
“...P_{index} as a proxy for desolubilization in three biological replicates at 4 time-points during germination, and in vegetative yeasts. In total, we…”

d. 2B. The authors need to indicate the identities of the genes in each of the groups shown. Table S4 could easily house this information. As it stands, it is difficult to interpret the data based on the handful of examples provided in Figure 3.

We modified our table to include the cluster attributed to each proteins

	Pindex					
Systematic name	0h	1h	3h	6h	vegetative cells	Cluster
YAL003W	0.33483348	0.25533162	0.31925034	0.46049299	0.315911422	Mostly in supernatant
YAL005C	0.21945126	0.18832197	0.18530921	0.64456277	0.200656213	Mostly in supernatant
YAL012W	0.04410389	0.07951884	0.03860619	0.06325469	0.069915642	Mostly in supernatant
YAL016W	0.61410372	0.37593279	0.244234	0.25185204	0.253032038	Gradual solubilization

“e. 2B: It would be helpful if the groups in the figure were referred to with the same nomenclature in the Figure Legend.”

This mistake was corrected. We uniformized the cluster names.

“f. 2C: The image in Figure 2C appears to be identical to the one shown on the left-hand side of Figure S2C. Was Figure S2C intended to plot PC2 against PC3?”

Principal component analysis was removed from Figure 2. The only occurrence of PC analysis is now in Figure S2.

“7) *Comments on Figure 3 data and results*

a. On Page 8 in the first half of the second paragraph the logic used by the authors is not clear. They indicate that there was no GO term enrichment for integral membrane proteins, so there must have been little membrane contamination in their preparations. Given that the preps were run on polyacrylamide gels prior to protein extraction, this seems like it should be the expectation. Perhaps they did not expect to detect lipid binding proteins and PI3P-binding proteins in the absence of membranes? Their explanation for the presence of these proteins is that there was a "gain of activity of many cellular pathways," which seems obvious, given that spores are germinating and becoming active. A more clear explanation of the expectations and outcomes here is warranted. It would also be useful to indicate explicitly why a protein like Sec18 (and its 16 companions) would show gradual insolubility when there is likely more vesicle trafficking activity.”

We explained the Pindex profiles of these proteins as follow in the text:
“For instance, gradual insolubility of the SNARE chaperone Sec18 may reflect increasing assembly of membrane-fusion complexes as vesicle transport is resumed to sustain cell growth”

“b. p. 8 second paragraph ". . . Ola1 and Yef3, which are known to aggregate in response to heat stress and disaggregate during recovery . . ."

We changed the sentence as suggested:
“…stress related proteins Ola1 and Yef3, which are known to aggregate in response to heat stress and disaggregate during recovery (Wallace et al., 2015).”

“c. Figure 3C: The presentations in the figure and the legend make these data confusing. (The text is more clear.) The legend states that Acc1 and Ura7 have similar behaviors as "they both condense upon stress." What's shown in the microscopy in Figure 3C is that they decondense during germination - supporting the idea that spores represent a stressed state that is relieved during germination. The legend needs to describe the discovery in this figure - not what was already known. Same for Glk1. In addition, the cartoon images shown above and below the microscopic images do not assist the reader. The images are not explained in the legend, and they are identical except for the addition of the word "relief." It is unclear what the little dots represent, what the green glob represents, and how they relate to the cells below and above. Is the dotted line representative of a cell outline? It seems like the top one is backwards, if the idea is to convey that germination likely represents stress relief.”

d. Figure 3D: The schematic of the model needs to be described more clearly in the figure legend and emphasize that the pink and blue assemblies represent

only the assemblies of enzymes that are needed for growth and metabolism. It should be noted that the enzyme assemblies for proteins no longer needed have the opposite behavior. (And the model is based on the behaviors of only 3 proteins.)

We agree that, as described, the schematics were confusing. We removed the schematics for clarity and changed the legend to better explain this discovery and the model.

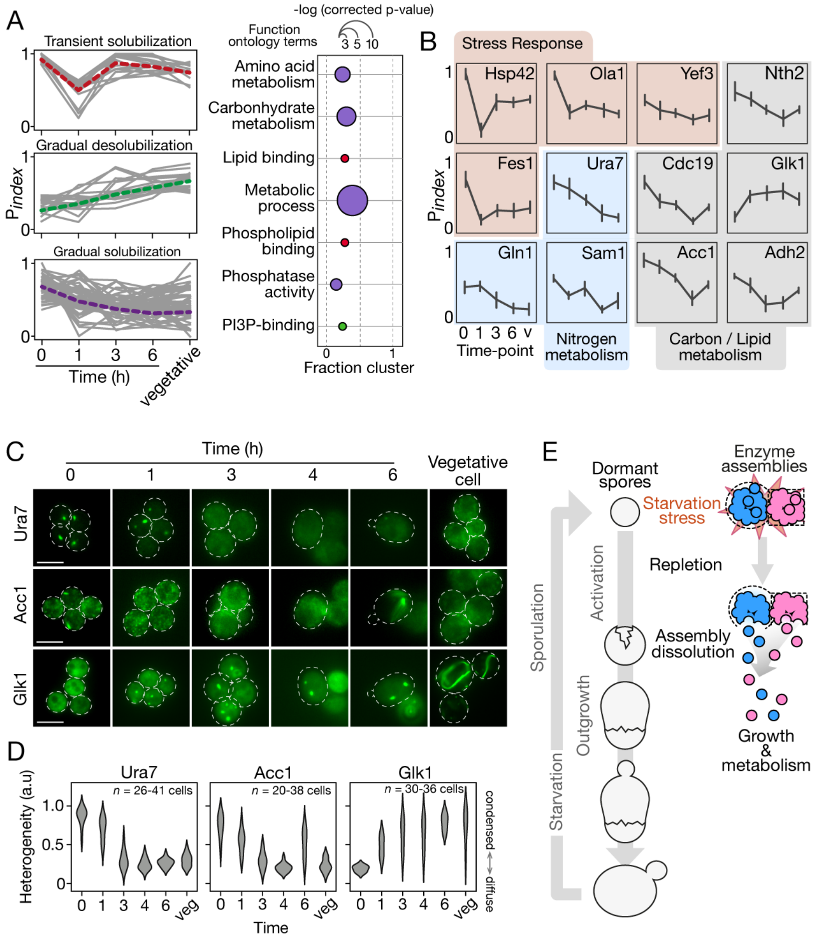

Figure 3 - Solubility changes reflect metabolism activation and mimic stress relief during germination.

A) Enrichment for gene ontology terms in each dynamically changing solubility cluster. Red, transiently solubilizing cluster; Green, gradual desolubilization cluster; Purple, gradual solubilization cluster. The position on the x-axis indicates the portion of the proteins in a cluster assigned to a GO term, and size of the bubble is scaled to the -log (*p*-values) from a hypergeometric test.

B) Individual P_{index} trajectory for representative proteins through germination. Bar represents standard variation. Proteins are clustered by function; Red, stress response proteins; Blue, Nitrogen metabolism proteins; Gray, Lipid and carbon metabolism proteins. Error bars represent standard deviation of three replicates.

C) Representative fluorescence microscopic images of spores expressing the indicated proteins tagged with GFP during germination. Top to bottom, Acetyl-CoA carboxylase Acc1 (Lipid

biosynthesis), CTP synthase Ura7 (pyrimidines synthesis) and Glucokinase Glk1 (glycolysis). The Glk1 foci formation and the dissolution of Acc1 and Ura7 foci in course of germination support that dormancy in spores is analogous to a stress state and germination alleviates this state. Dotted lines indicate cell contour determined by brightfield images. Scale bars represent 5µm.
D) Measure of cellular heterogeneity (coefficient of variation) of the fluorescent proteins in spore at the indicated time after exposure to rich medium or in vegetative cells. Between 20 and 41 cells were analyzed at each time-points.
E) Schematics highlighting effects on protein solubility of nutrient starvation and repletion during sporulation and germination, respectively. Pink and blue assemblies represent assemblies of enzymes needed for growth and metabolism during dormancy, which disassemble (pink and blue circles) during germination.

"8) Comments on Figure 4 data and results
a. The authors need to make it more clear what was known prior to this study and what they discovered here. They need to be conservative in their interpretations of the phospho-proteome data because of the limited (although expected) number of peptides detected. Given that Hsp42 was the only protein with the transient solubilization profile and a phosphoprotein hit, it is important that the authors provide the identities of the other 14 proteins in the transient solubilization group so that they can be evaluated."

We agree on this point. Therefore, identities of the 15 proteins in the Transient solubilization cluster are shown in Figure S5C

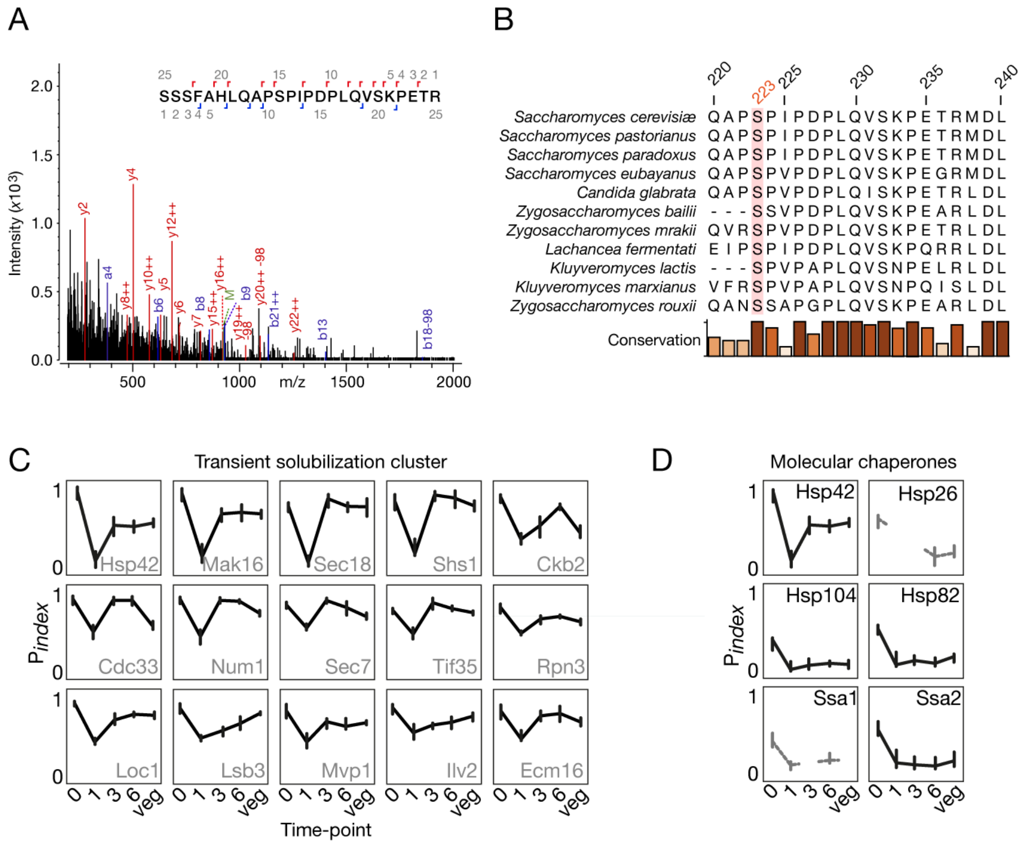

“9) a. The explanation for the rationale and results for Figure 5 are not clear. The top paragraph of p. 11 needs to establish what was known previously, convey the hypothesis that the authors were testing, and explain their new discoveries.”

Last section of the results section entitled “**Hsp42 activity is crucial for normal progression of germination”** was rewritten to include and discuss the results about trehalose content in spores.

“b. The legend to Figure 5 needs to be written with proper genotypes.”

As suggested, the legend to Figure 5 was modified:

Figure 5 - Active and phosphorylated Hsp42 is required for normal germination dynamics.

A) Optical density of pure spore cultures of the indicated strains as a function of time following exposure to germination conditions. Shown are the mean values of three replicates. OD drop of the Δhsp42 spores is strongly delayed, indicating that germination is inhibited or slowed down. Spores expressing S223A Hsp42 mutant show a slight delay in germination, while germination of spores expressing S223E Hsp42 mutant is indistinguishable from that of wild spores.

B) Ensemble mean square displacement of µNS-GFP in WT and Δhsp42 spores at the indicated time after exposure to rich medium. At each time point for each strain, 25 to 35 particles, corresponding to the same number of cells, were tracked. Kruskal-Wallis test, ** indicates p-value < 0.0001, * indicates p-value < 0.01.
C) Fluorescence microscopy images of wild type (top) or ΔHsp42 spores expressing Acc1-mCherry at the indicated time after exposure to germination conditions. Scale bar represents 5µm.
D) Cellular Acc1-mCherry heterogeneity score in WT or Δhsp42 spores at the indicated time after exposure to rich medium.
E) Trehalose content (measured in equivalent of glucose concentration) in spores at the indicated time after exposure to rich medium, and in vegetative yeasts. Error bars represent standard deviation of three replicates.
F) Fluorescence microscopy images of spores expressing either WT, S223A or S223E mutant Hsp42-GFP and Acc1-mCherry at the indicated time after exposure to germination conditions. Dotted lines represent cell contour. Scale bar represents 5 µm. Expression of S223A Hsp42-GFP mutant shows delay in Acc1 foci dissolution in spores.

"In the Results section, please use the past tense for current discoveries and present tense for previously published data."

Thanks for that comment. We made sure to use the appropriate verb tense throughout the manuscript.

"p. 3 paragraph 2 ". . . germination coordinates the breaking of dormancy . . ."
p. 4 paragraph 2 ". . . biophysical properties of dormant budding yeast spores and the changes that occur during dormancy breaking to unveil the molecular processes that support . . .
p. 4 paragraph 2 ". . . changes taking place in spores mimic what occurs in yeast experiencing stress relief . . ."
p. 4 paragraph 2 the use of the word "implication" generally requires referral to a specific process or event. ". . . is the implication of a small heat shock protein, Hsp42 in spore germination, which is essential for . . ."
p. 5 paragraph 1 "Spores' transitions from high to low refractility correlate with a decrease in . . ."
p. 5 paragraph 1 "Taken together, these measurements . . . major time points that can be used to . . ."
p. 6 paragraph 2 "To test this hypothesis, we constitutively expressed the pH . . ."
p. 8 top paragraph 1 The use of "for instance" throughout the manuscript is unnecessary.
p. 8 bottom paragraph change "comportment" to "behavior"
Figure 3 legend: change "comportement" to "behavior"

These mistakes were all corrected
